# Antibody-guided identification of *Achromobacter xylosoxidans* protein antigens in cystic fibrosis

**DOI:** 10.1128/msphere.00233-25

**Published:** 2025-04-29

**Authors:** Cecilia Sahl, Sounak Chowdhury, Johan Malmström, Lisa I. Påhlman

**Affiliations:** 1 Division of Infection Medicine, Department of Clinical Sciences, Lund University 5193, , Lund, Sweden; 2 Wallenberg Centre for Molecular Medicine, Lund University 5193, Lund, Sweden; 3 Division of Infectious Diseases, Skåne University Hospital Lund 59564, Lund, Sweden; The University of Texas Medical Branch at Galveston, Galveston, Texas, USA

**Keywords:** *Achromobacter xylosoxidans*, cystic fibrosis, IgG antigens, systems antigenomics

## Abstract

Persistent bacterial airway infection is a hallmark feature of cystic fibrosis (CF). *Achromobacter* spp. are gram-negative rods that can cause persistent airway infection in people with CF (pwCF), but the knowledge of host immune responses to these bacteria is limited. The aim of this study was to investigate if patients develop antibodies against *Achromobacter xylosoxidans*, the most common *Achromobacter* species, and to identify the bacterial antigens that induce specific IgG responses. Seven serum samples from pwCF with *Achromobacter* infection were screened for antibodies against bacteria in an ELISA coated with *A. xylosoxidans*, *A. insuavis,* or *Pseudomonas aeruginosa*. Sera from pwCF with or without *P. aeruginosa* infection (*n* = 22 and 20, respectively) and healthy donors (*n* = 4) were included for comparison. Serum with high titers to *A. xylosoxidans* was selected for affinity purification of bacterial antigens using serum IgGs bound to protein G beads. The resulting IgG-antigen complexes were then analyzed using liquid chromatography-tandem mass spectrometry (LC-MS/MS). Selected antigens of interest were produced in recombinant form and used in an ELISA to confirm the results. Four of the seven patients with *Achromobacter* infection had serum antibodies against *Achromobacter*. Using patient serum-IgG for affinity purification of *A. xylosoxidans* proteins, we identified eight antigens. Three of these, which were not targeted by anti-*P*. *aeruginosa* antibodies, were expressed recombinantly for further validation: dihydrolipoyl dehydrogenase (DLD), type I secretion C-terminal target domain-containing protein, and domain of uncharacterized function 336 (DUF336). While specific IgG against all three recombinant antigens was confirmed in the patient serum with high titers against *Achromobacter*, DLD and DUF336 showed the least binding to serum IgG from pwCF without *Achromobacter* spp. infection. Using serum IgG affinity purification in combination with LC-MS/MS and confirming the results using ELISA against recombinant proteins, we have identified bacterial antigens from *A. xylosoxidans*.

**IMPORTANCE:**

*Achromobacter* species are opportunistic pathogens that can cause airway infections in people with cystic fibrosis. In this patient population, persistent *Achromobacter* infection is associated with low lung function, but the knowledge about bacterial interactions with the host is currently limited. In this study, we identify protein antigens that induce specific antibody responses in the host. The identified antigens may potentially be useful in serological assays, serving as a complement to culturing methods for the diagnosis and surveillance of *Achromobacter* infection.

## INTRODUCTION

Cystic fibrosis (CF) is a monogenic disease caused by mutations in the CF transmembrane regulator (CFTR) gene. These mutations result in impaired chloride transport across cell membranes, leading to the accumulation of abnormally thick mucus in the airways and pancreatic ducts. The viscous CF mucus provides a favorable environment for bacterial infections. Persistent respiratory tract infections and the resulting airway inflammation cause progressive lung injury, ultimately leading to respiratory failure, which is the primary cause of mortality in this patient population (1).

*Achromobacter* spp. is a genus of environmental gram-negative bacteria capable of causing opportunistic airway infections in people with CF (pwCF) (2, 3). The prevalence of *Achromobacter* spp. infection in pwCF is approximately 5%, although the rate varies between countries (4). *Achromobacter xylosoxidans* is the most frequently identified *Achromobacter* species in pwCF, but other species, including *A. insuavis*, are also reported (5, 6). Persistent *Achromobacter* spp. infection is associated with decreased lung function (7), an increased need for intravenous antibiotics (8), more frequent pulmonary exacerbations, and a higher risk of requiring lung transplantation (9). However, it remains unclear whether *Achromobacter* actively contributes to these outcomes or if its presence is merely indicative of low lung function caused by other factors (3, 10).

While common CF pathogens such as *Pseudomonas aeruginosa* are well-characterized, the virulence mechanisms and host responses to *Achromobacter* are less understood. According to a previous study, pwCF infected with *Achromobacter* develop antibody responses against the bacteria (11). However, the specific antigenic targets are not yet identified. The aim of this study was to identify proteins expressed by *Achromobacter* that are recognised by host antibodies. To characterize *Achromobacter* antigens targeted by circulating IgGs in pwCF, we took advantage of a novel mass spectrometry-based systems antigenomics approach (12) where serum IgG from pwCF infected with *Achromobacter* spp. were used to affinity-purify bacterial antigens from the most common species of *Achromobacter,* that is, *A. xylosoxidans*.

## MATERIALS AND METHODS

### Human sera and bacterial isolates

Serum samples were collected from adult people with CF attending the CF center at Skåne University Hospital, Lund, Sweden. Individuals with repeated growth of *Achromobacter* spp. in airway cultures were regarded to have *Achromobacter* airway infection. Chronic *P. aeruginosa* infection was defined as growth in ≥50% of airway cultures according to Leeds criteria (13). Sera from healthy individuals were included as controls.

*A. xylosoxidans* type strain 56,438T and *A. insuavis* type strain 62,426T were purchased from the Culture Collection University of Gothenburg, Sweden. The *P. aeruginosa* reference strain PAO1 was a gift from Professor Arne Egesten, Lund University. Clinical *Achromobacter* spp. isolates were obtained from the Department of Clinical Microbiology at Skåne University Hospital, Lund, Sweden. Species identification of clinical *Achromobacter* isolates was done using standard laboratory methods at the Department of Clinical Microbiology at Skåne University Hospital, Lund, Sweden, followed by *nrdA* sequencing as described previously (14).

### ELISA of serum IgG binding to whole bacteria

Three to four colonies of *A. xylosoxidans*, *A. insuavis*, or *P. aeruginosa* type strains were suspended in 20 mL coating buffer (1.69 g Na_2_CO_3_ and 2.94 g NaHCO_3_ in 1 L H_2_O, pH 9.6) and dispensed at a volume of 200 µL into a 96-well microtiter plate (Nunc Maxisorp). After incubation overnight at 37°C, 5% CO_2_, the coating buffer was removed, and bacteria were heat-killed at 80°C for 15 min. Unspecific binding was blocked by incubating with 5% BSA in PBST (blocking buffer) for one hour. Sera were diluted 1:100 in blocking buffer, added at a volume of 200 µL to the coated wells, and incubated for 1 h. The plates were then washed three times with PBST. Protein G-HRP conjugate (Bio-Rad #170-6425) was diluted 1:3,000 in blocking buffer and added at a volume of 200 µL. The plates were incubated for 1 h, after which the wash step was repeated. A total of 200 µL of developing solution (600 µL of 2% [wt/vol] ABTS and 248 µL 0.6% H_2_O_2_ in 12 mL of substrate buffer (0.1 M C_6_H_8_O_2_ × H_2_O) and 0.1 M Na_2_HPO_4_ × 2H_2_O, pH 4.5) was added and incubated in the dark for 5 min. The absorbance was read at 420 nm, and the background OD of blank wells was subtracted. The average OD value of the healthy control group + 3 standard deviations was considered the cut-off for positivity.

### Preparation of bacterial secreted and surface proteins

*A. xylosoxidans* was grown overnight in ABT medium (15) supplemented with 0.5% (wt/vol) of casamino acids and 0.5% (wt/vol) glucose (Merck). The following day, a subculture with OD_620_ = 0.1 was started in the same medium and grown to the mid-logarithmic phase (OD_620_ ≈ 0.4) with shaking incubation at 37°C and 5% CO_2_. The resulting culture was transferred to 50 mL tubes and centrifuged at 10,000 × *g*, 30 min, at 4°C.

Secreted proteins were prepared from the supernatant. After sterilization using a 0.22 µm filter (Merck-Millipore #SLGP033RB), the supernatant was purified through 10 kDa ultrafiltration columns (Merck-Millipore #UFC901008) by centrifuging at 4,000 × *g* for 12 min at 4°C. The fraction collected in the filter was washed three times with ice-cold PBS and saved.

Surface proteins were prepared from the bacterial pellet. After washing two times in HEPES buffer, bacteria were resuspended in HEPES buffer at a concentration of approx. 1 × 10^8^ CFU. A total of 0.2 µg trypsin (Promega V5113) per mL of original bacterial culture was added and incubated for 60 min at 37°C, 500 rpm. The reaction was then stopped by incubating on ice for 2 min. The samples were centrifuged at 1,000 × *g* for 15 min at 4°C, and the supernatant was transferred into new tubes. The resulting surface protein fraction was incubated for 15 min at 85°C, 500 rpm to heat-kill any remaining bacteria.

Protein concentrations in both fractions were measured using the Pierce BCA Protein Assay Kit (Thermo Scientific, 23225) according to the manufacturer’s instructions, after which the fractions were saved at −20°C.

### Western blot

Bacterial protein fractions were mixed 4:1 with 5× loading buffer (GenScript #MB01015) and denatured at 85°C for 10 min before loading onto an SDS-PAGE gel (Mini-PROTEAN TGX Precast, Bio-Rad) and run at 170 V for 1 h. The gel was blotted onto the membrane (Bio-Rad Trans-Blot Turbo #1704156) and blocked with 5% BSA in PBST for 1 h. The membrane was washed three times with PBST and incubated overnight at 4°C with 1:10,000 human serum in blocking buffer. After washing, the membrane was incubated with protein G-HRP conjugate diluted 1:3,000 in blocking buffer for 1 h at 37°C. The membrane was washed as described and then developed using Clarity Western ECL Substrate (Bio-Rad #1705061) according to the manufacturer’s instructions. Images were taken using the ChemiDoc (Bio-Rad).

### Affinity purification of bacterial antigens

The affinity purification was performed essentially as described by Chowdhury et al. 12. Bacterial proteins were affinity purified using IgG from the following sources: one serum from pwCF infected with *A. xylosoxidans* (CF_Achro_), one serum from pwCF infected with *P. aeruginosa* (CF_PsA_), one healthy control serum, omalizumab (Xolair, Novartis), and pharmaceutical grade intravenous immunoglobulin G (IVIG, Octagam Octapharma). Omalizumab and IVIG were used at a concentration of 1 mg/mL, and the serum samples were diluted 1:10 to approximately match this concentration.

A total of 50 µL Pierce Protein G magnetic beads (ThermoFisher #13424229) was added to 1.5 mL microcentrifuge tubes. The beads were washed three times with 200 µL TBST using a magnetic rack. In total, 100 µL of each immunoglobulin sample and 400 µL TBST were added to the vials in triplicates and incubated with shaking at room temperature for 1 h. The beads were then washed with 500 µL TBST three times. The secreted and surface fractions of *Achromobacter* proteins were pooled so that 500 µL added to the beads contained 25 µg of each fraction, and the samples were then incubated at room temperature overnight. The beads were then washed three times with TBST before eluting the IgG-antigen complexes with 100 µL 0.1 M glycine. To neutralize the pH, 15 µL of 1 M Tris was added. Next, 15 µL of the eluted samples were run on SDS-PAGE as described above, and the gel was stained overnight with PageBlue solution (ThermoFisher #24620) to confirm the presence of antibody complexes. All affinity purifications were performed in triplicates.

### Sample preparation for mass spectrometry and liquid chromatography-tandem mass spectrometry (LC-MS/MS) analysis

Samples obtained from affinity purifications as well as samples from both bacterial protein fractions were homogenized at 4,350 rpm for 90 s (BeadBug 6, Benchmark Scientific). Fifty microliters of each sample was denatured in 50 µL of ABC-urea buffer (8 M urea, 100 mM ammonium bicarbonate in HPLC-grade H_2_O). To reduce disulfide bonds, 0.5 µL of 500 mM Tris(2-carboxyethyl)phosphine was added, and the samples were incubated at 37°C for 1 h. Subsequently, 1 µL of 500 mM 2-iodoacetamide was added to alkylate the disulfide bonds, followed by a 30-min incubation at room temperature in the dark. Samples were incubated at 37°C overnight with 2 µL of 0.5 µg/µL trypsin. The reaction was then stopped by adding formic acid until pH 3.

Harvard apparatus Macro SpinColumns Silica C18 were used for sample purification according to the manufacturer’s instructions. The eluate was concentrated by SpeedVac (ThermoFisher) until complete dryness and resuspended in 20 µL 0.1% formic acid. Resuspended samples were sonicated for 5 min and transferred to MS vials, which were stored at −20°C until analysis.

The peptides were analyzed using a Q Exactive HFX instrument (Thermo Scientific) coupled to an Easy-nLC 1200 system (Thermo Scientific), in data-dependent acquisition (DDA) mode LC-MS/MS as described in Chowdhury et al. (12). Samples were picked up by the Easy-Spray LC column (50 cm, 45°C, Thermo Scientific) at a volume of 2 µL with 20 µL flow, followed by loading 6 µL with 5 µL/min flow. The maximum pressure was set to 800 bar. A linear gradient of 4–45% acetonitrile in 0.1% formic acid was run for 65 min. One full MS scan (resolution of 60,000 for a mass range of 390–1,210, automatic gain control = 3e6) was followed by MS/MS scans (resolution of 15,000, automatic gain control = 1e5) of the 15 most abundant signals. Isolation width was set to 2 *m/z* for precursor ions, and fragmentation was performed with higher-energy collisional-induced dissociation at a normalized collision energy [(N)CE] of 30.

### Data analysis and statistics

The DDA data were analyzed in MaxQuant (16) (version 2.2.0.0) using the *A. xylosoxidans* reference proteome (Uniprot proteome ID: UP000595052) with the addition of common contaminants and RT peptides to the reference proteome file. Oxidation (M) and acetyl (protein N-term) were used as variable modifications. Carbamidomethyl (C) was used as a fixed modification. The maximum number of modifications per peptide was 5. Fast LFQ was used with a minimum ratio count of 2, a minimum of 2, and an average of 6 neighbours. Identification was performed with a false discovery rate (FDR) of 0.01 and matching between runs.

The data set obtained was further filtered in Perseus (17) (Cox Lab, version 2.0.7.0). Data were filtered to remove those identified by site, reverse, and potential contaminants. Hits with <2 unique peptides or present in <2 of the triplicates were excluded. After filtering and before statistical analysis, the LFQ intensities were log2 transformed and missing values replaced using imputed values from normal distribution. Proteins were considered antigenic hits if they were significantly enriched in the CF_Achro_ serum compared to the negative control omalizumab (double-sided *t*-test, FDR 0.05).

### Recombinant protein expression and ELISA of serum IgG binding to recombinant proteins

The amino acid sequences of three putative antigens (dihydrolipoyl dehydrogenase [DLD; Uniprot identifier A0A0D6GPL2], type I secretion C-terminal target domain-containing protein [T1S-DCP; A0A7T2RJE8], and domain of uncharacterized function 336 [DUF336; A0A0D6GMC1]) were submitted to Protein Production Sweden for recombinant expression of proteins in *Escherichia coli*. Protein suspensions were diluted to a concentration of 4 µg/mL (T1s_DCP and DUF336) or 2 µg/mL (DLD) in coating buffer as described earlier. The coating buffer for the T1S protein was supplemented with 5 mM calcium chloride dihydrate. The ELISA was performed in triplicates as described above, except for the serum concentration being 1:50 and the development time for the colorimetric readout being 30 min.

### PCR

Bacterial colonies were resuspended in PBS and placed in a heat block at 100°C for 5 min. Samples were spun down at 12,000 × *g* for 5 min and the resulting supernatants were used as PCR template in 1:10 dilution. Conventional PCR was performed with 1 U Taq-Polymerase “Phusion HS-II” (Thermo Scientific) and 0.5 µM primers (T1S-DCP forward CGAGGCCTACCTGAAGTTCTTT, T1S-DCP reverse CCATCGGTGTTGAGGGTGTC, DUF336 forward ACAAGTGGGCTGTCACCATC, DUF336 reverse CCGACAGGAAGGAATAGCGG, DLD forward TTCACCAAGCAAGGCCTGAA, DLD reverse CACGGCTTCCGAGATCAGTT) per 2 µL DNA sample. PCR products were visualized on 1% agarose (Thermo Scientific) using gel electrophoresis.

### Local alignment search (basic local alignment search tool [BLAST])

BLAST in protein-protein mode was used to identify sequence similarity of *A. xylosoxidans* DLD, T1S-DCP, and DUF336 in comparison to *A. insuavis* (taxid 1287735) and *P. aeruginosa* (taxid: 287). The search was performed with the standard database of non-redundant protein sequences and scored with the BLOSUM62 matrix. The protein with the highest similarity in each comparison was selected and presented in File S3. Multiple sequence alignment was performed using CLUSTALW alignment with the weight matrix BLOSUM for proteins, and the alignment score was calculated by CLUSTALW from the number of identities between the two sequences divided by the length of the alignment.

### Statistics

Enrichment analysis for antigenic hits was performed using volcano plots in Perseus 2.0.7.0 (double-sided *t*-test, FDR 0.05). Statistical calculations for ELISA experiments were performed using GraphPad Prism 10.0.2 (GraphPad Software, San Diego, CA, USA). Comparisons between groups were made using the non-parametric Mann-Whitney *U*-test for continuous variables. A *P* value ≤ 0.05 was considered statistically significant.

## RESULTS

### *Achromobacter* infection evokes circulating antibodies in pwCF

**Fig 1:**
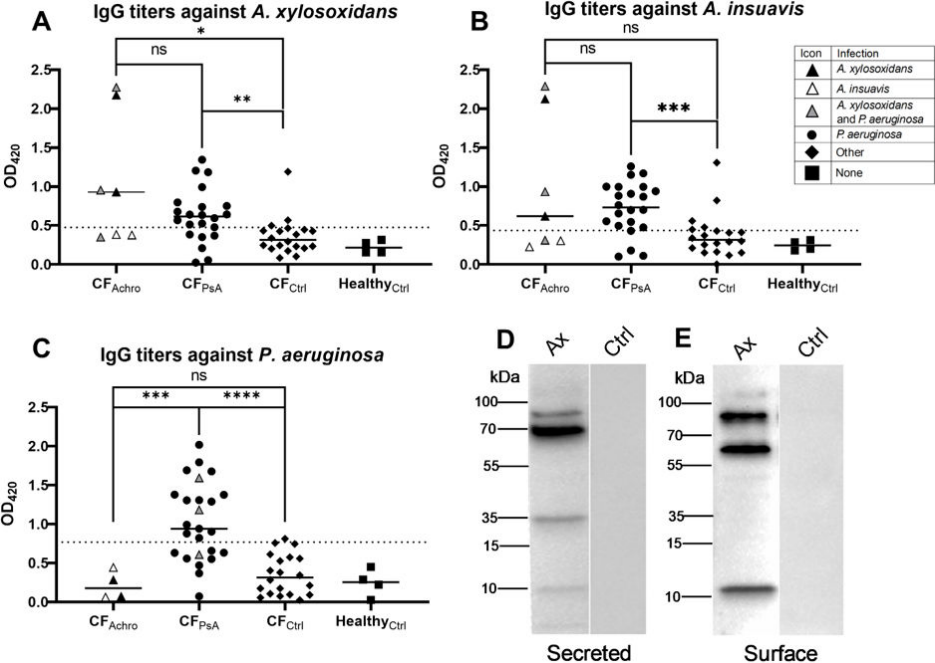
Antibody responses of donor serum against whole bacteria and *Achromobacter* proteins. Serum samples from pwCF and healthy controls were analyzed for IgG titers against *Achromobacter xylosoxidans* (**A**), *A. insuavis* (**B**), and *Pseudomonas aeruginosa* (**C**) by direct ELISA using plates coated with whole bacteria. Bars represent the mean OD_420_ value per group. Each dot is the average value of three technical replicates. Serum from individuals infected with both *Achromobacter* and *P. aeruginosa* (gray triangles) was included in the *Achromobacter* group when screening for those antibodies (**A, B**), and vice versa for *P. aeruginosa* (**C**). The dotted lines represent the average of the healthy control group +3 SD, which was considered the cutoff value for positive titers. Groups were compared using the Mann-Whitney test. (**D, E**) Western blot assay of serum IgG binding to *A. xylosoxidans* proteins. Secreted proteins (**D**) and trypsinated surface fractions (**E**) were separated on SDS-PAGE followed by Western blotting using CF_Achro_ and healthy control serum.

To evaluate IgG responses against *Achromobacter* spp., serum samples from seven pwCF with *Achromobacter* spp. airway infection (CF_Achro_) were screened for antibody titers against whole *Achromobacter* bacteria. Five of the seven individuals were infected with *A. xylosoxidans* and two with *A. insuavis*. Three of the patients were also chronically co-infected with *P. aeruginosa*. To quantify serum-IgG against whole bacteria, microtiter plates were coated with either *A. xylosoxidans* or *A. insuavis,* and serum IgG titers were then analyzed by ELISA. For comparison, serum samples from pwCF with chronic *P. aeruginosa* infection (CF_PsA_, *n* = 22), sera from pwCF controls without *Achromobacter* or *P. aeruginosa* infection (CF_Ctrl_, *n* = 20), and sera from healthy donors (*n* = 4) were included in the analysis. Four of the seven individuals infected with *Achromobacter* spp. (57%) had positive serum titers against both *A. xylosoxidans* and *A. insuavis* (Fig. 1A and B). All four individuals were infected with *A. xylosoxidans*. In contrast, none of the two study participants infected with *A. insuavis* had serum IgG against either of the two *Achromobacter* species. As a group, the CF_Achro_ sera had higher IgG titers against *A. xylosoxidans* compared to CF_Ctrl_ (*P* = 0.02, Fig. 1A), whereas the difference in titers against *A. insuavis* was not significant between the CF_Achro_ and CF_Ctrl_ groups (*P* = 0.07, Fig. 1B). Surprisingly, CF_PsA_ sera had significantly higher median titers than CF_Ctrl_ sera against both *A. xylosoxidans* (*P* = 0.002, Fig. 1A) and *A. insuavis* (*P* = 0.0007 Fig. 1B). Therefore, to test whether *Achromobacter* infection triggers antibodies reactive against *P. aeruginosa*, all sera were also screened for IgG titers against *P. aeruginosa*. A majority of the CF_PsA_ group (64%) had IgG titers against *P. aeruginosa* (Fig. 1C). However, CF_Achro_ patients did not have serum IgG against *P. aeruginosa,* except for three patients who were infected with both *Achromobacter* and *Pseudomonas*.

Since only patients infected with *A. xylosoxidans*, but not *A. insuavis*, raised IgG responses against *Achromobacter* spp., we wanted to identify *A. xylosoxidans* protein antigens responsible for inducing adaptive immunity in these individuals. Since the IgG responses observed in Fig. 1A were directed against whole bacterial cells, we prepared secreted and surface fractions of *A. xylosoxidans* for the identification of specific protein antigens (Fig. S1). These protein fractions were separated by SDS-PAGE, followed by Western blotting using serum from a patient in the CF_Achro_ group with high titers against *A. xylosoxidans*. The results demonstrated multiple bands both in the secreted and surface fractions, indicating IgG-binding to bacterial antigens (Fig. 1D and E). In contrast, no IgG binding was detected with serum from a healthy control (Fig. 1D and E). This qualitative screening suggests that *A. xylosoxidans* infection results in circulating antibodies against various *A. xylosoxidans* secreted and surface proteins.

### Identification of *Achromobacter* protein antigens

**Fig 2:**
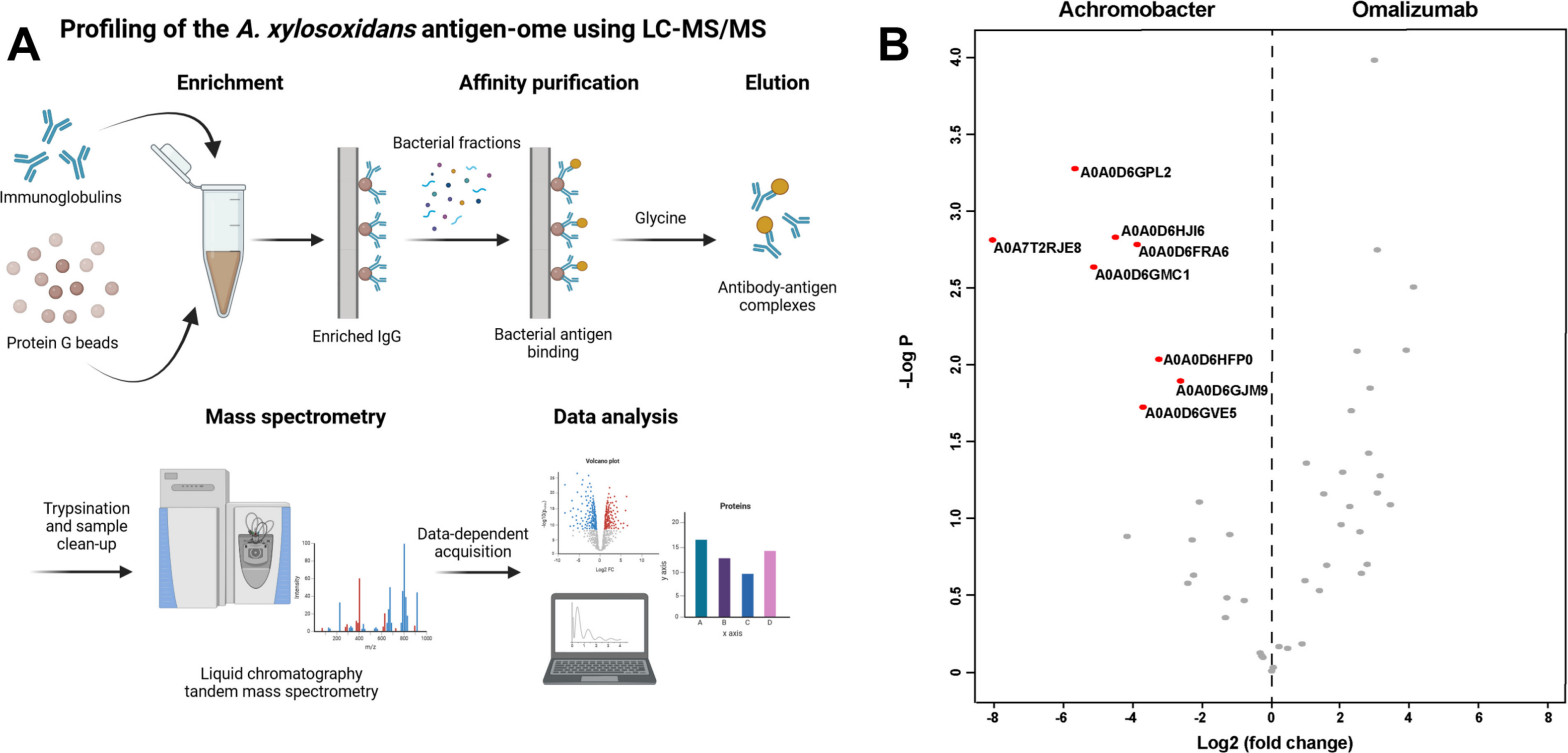
Identification of antigens in *Achromobacter xylosoxidans*. (**A**) Brief description of the systems antigenomics workflow used to identify antigenic proteins. (**B**) Enrichment analysis of bacterial proteins associated with serum from pwCF infected with *Achromobacter* compared to omalizumab, two-tailed *t*-test, FDR 0.05. Panel A created with BioRender.com.

Having confirmed that *A. xylosoxidans* airway infection can give rise to circulating antibodies against *Achromobacter*, we next aimed to quantitatively identify these bacterial antigens. LC-MS/MS analysis of the secreted and surface fractions of *Achromobacter* identified a total of 357 bacterial proteins in the secreted fraction and 1,163 proteins in the surface fraction (File S1). Flagellin was the most abundant protein in both fractions, followed by T1S-DCP in the secreted fraction and chaperonin in the surface fraction. To quantitatively identify *Achromobacter* antigens triggering an antibody response, we took advantage of a newly established mass spectrometry-based systems antigenomics workflow (12), wherein secreted and surface fractions were pooled and passed over immobilized IgG from serum of different sources. The affinity purification resulted in antigen-antibody complexes, which were then eluted by glycine and analyzed using LC-MS/MS (Fig. 2A; File S2). Serum from an individual with high IgG titers against *A. xylosoxidans* (CF_Achro_ serum) was used in this experimental setup. To control for non-specific IgG binding, the monoclonal anti-IgE antibody omalizumab was included as a negative control. When comparing proteins enriched from IgG pulldowns using CF_Achro_ serum to those from the negative control omalizumab, eight proteins were found to be significantly associated with IgG from CF_Achro_ serum (Fig. 2B; File S2), suggesting that *Achromobacter* infection triggers an antibody response against a small subset of proteins. These antigens were predominantly present in both secreted and surface fractions and were involved in different cellular functions such as iron binding, enzymatic activity, and membrane transport (Table 1). To further determine that the antibody response against these antigens was a result of *Achromobacter* infection, we also performed the analysis on pooled IgGs from multiple individuals (IVIG) and serum from a healthy donor. IVIG and pooled human plasma were considered negative controls as *Achromobacter* is an opportunistic pathogen that rarely causes infection in healthy individuals. All eight *A. xylosoxidans* antigens identified with CF_Achro_ serum were absent in the IgG pulldowns using IVIG and healthy donor serum (Fig. S2), further supporting that the IgG response induced by *Achromobacter* infection is highly specific.

**TABLE 1:** *A. xylosoxidans* antigens

UniProt ID	Name	Fraction	Function
A0A7T2RJE8	Type I secretion C-terminal target domain-containing protein	Secreted, surface	Membrane transport
A0A0D6GPL2	Dihydrolipoyl dehydrogenase	Secreted, surface	Dehydrogenase activity
A0A0D6GMC1	Domain of uncharacterized function (DUF336)	Secreted, surface	Heme binding
A0A0D6HJI6	DNA protection during starvation protein 2	Surface	Iron binding
A0A0D6FRA6	Amino acid ABC transporter substrate-binding protein	Secreted, surface	Membrane transport
A0A0D6GVE5	Inorganic pyrophosphatase	Secreted, surface	Phosphatase activity
A0A0D6HFP0	Glutamate dehydrogenase	Secreted, surface	Dehydrogenase activity
A0A0D6GJM9	Alkyl hydroperoxide reductase AhpD	Secreted, surface	Hydroperoxidase activity

### Specificity of identified antigens to *A. xylosoxidans*

**Fig 3:**
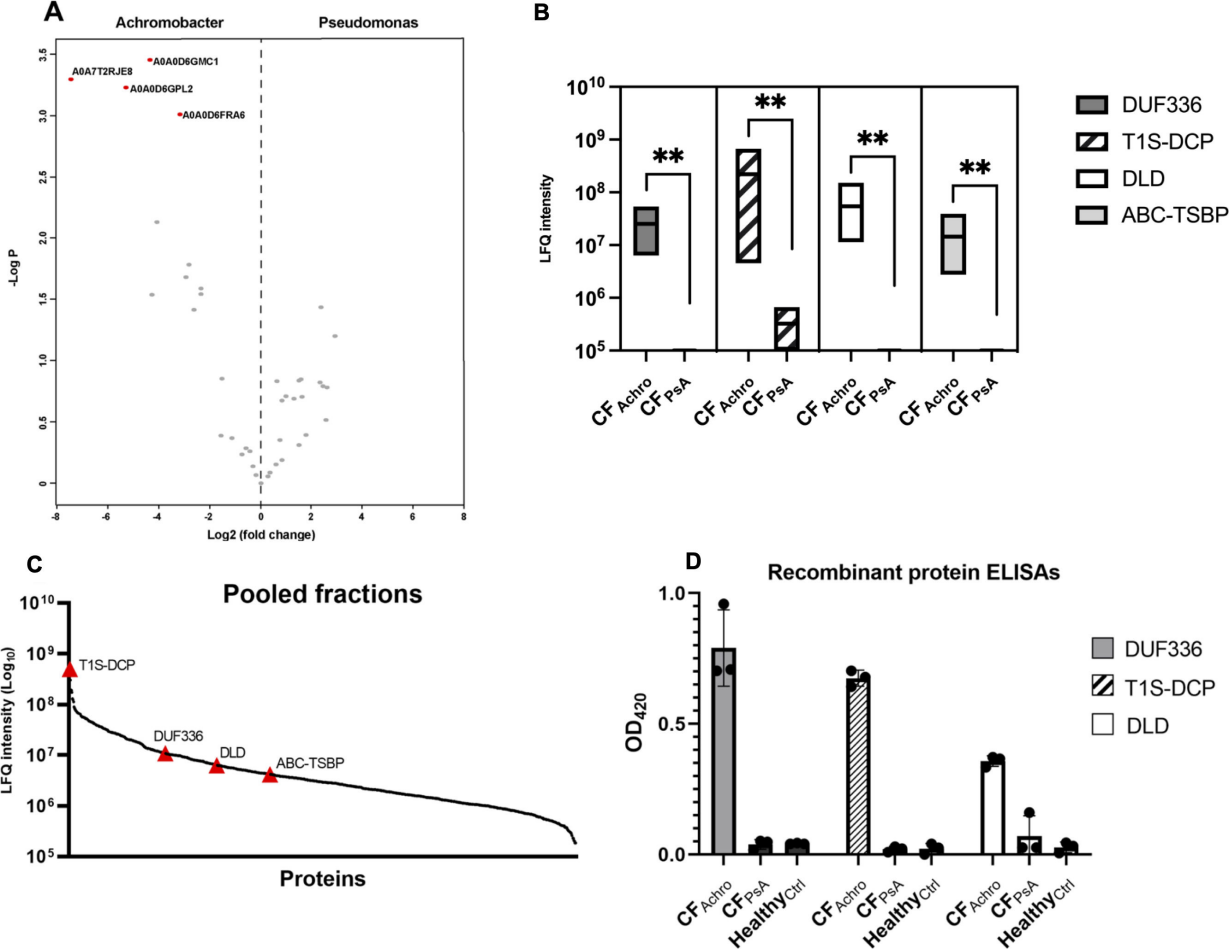
Specificity of Achromobacter antigens compared to anti-P. aeruginosa IgG. (**A**) Enrichment analysis of bacterial proteins associated with CF_Achro_ serum compared to CF_PsA_ serum, two-tailed *t*-test, FDR 0.05. (**B**) LFQ intensities of the Achromobacter antigens DUF336, T1S-DCP, DLD, and ABC-TSBP in pulldowns using CF_Achro_ and CF_PsA_ serum are compared using the Mann-Whitney *U*-test. *n* = 6, ** = *P* < 0.01. (**C**) Abundance of significant Achromobacter antigens in the total bacterial protein fraction. LFQ intensities (log10) of all 935 identified Achromobacter proteins in the pooled bacterial protein fraction are plotted in descending order. The four CF_Achro_-associated bacterial antigens are marked with red triangles. (**D**) ELISA plates were coated with recombinantly expressed antigens, and IgG titers were tested in the same sera used in the IgG pulldown assay.

To rule out antigens which may also be targeted by *P. aeruginosa* antibodies, we also performed IgG affinity purification of *Achromobacter* proteins using IgG derived from an individual with *P. aeruginosa* infection who had high antibody titers against *A. xylosoxidans* (CF_PsA_ serum, Fig. 1A). This individual was not co-infected with *A. xylosoxidans* and had no known history of *Achromobacter* infection. When comparing intensities of *A. xylosoxidans* antigens purified with IgG derived from CF_Achro_ and CF_PsA_ serum respectively, four antigens were significantly associated with CF_Achro_ serum compared to CF_PsA_ serum (Fig. 3A). These proteins were DLD (A0A0D6GPL2), T1S-DCP (A0A7T2RJE8), DUF336 (A0A0D6GMC1), and amino acid ABC transporter substrate-binding protein (ABC-TSBP, A0A0D6FRA6). Additional repeats of the IgG affinity purification confirmed that all four antigens had significantly higher intensities in pulldowns using CF_Achro_ serum, and three of the antigens (DLD, DUF336, and ABC-TSBP) were not identified at all in pulldowns using CF_PsA_ serum (Fig. 3B). The four antigens were distributed across the intensity range of proteins identified from the pooled bacterial fractions (Fig. 3C), suggesting that the affinity purification was not biased towards highly abundant proteins.

To further validate the IgG pulldown results, we selected three antigens, i.e., DLD, T1S-DCP, and DUF336, that were recombinantly expressed in *E. coli*. The recombinant proteins were used to coat ELISA plates, after which the patient and control sera used in the affinity purification protocol were added to quantify IgG binding. In agreement with the IgG pull-down results, we observed high IgG titers to all these three recombinant proteins in the CF_Achro_ serum, whereas the healthy donor serum and CF_PsA_ serum did not have IgG titers against any of the three antigens (Fig. 3D). Taken together, the ELISA results with recombinant antigens confirmed the identification of specific *A. xylosoxidans* antigens using the MS-based systems antigenomics workflow.

### Evaluation of *A. xylosoxidans* antigens in a wider CF cohort

To further investigate the specificity of *A. xylosoxidans* antigens, we performed a BLAST search and amino acid sequence alignment of the three antigens to identify similar proteins in *A. insuavis* and *P. aeruginosa*. Using a cutoff of 50% alignment score to define protein homology, DUF336 showed a high similarity to heme-binding proteins in *A. insuavis* (sequence homology 97%) and in *P. aeruginosa* (56.8%). DLD was found in both species and was considered homologous to *A. xylosoxidans* DLD in *P. aeruginosa* (68%) but not in *A. insuavis* (31.9%) (File S3). No homologous proteins to T1S-DCP were identified in either *A. insuavis* or *P. aeruginosa*.

We further aimed to confirm that DLD, T1S-DCP, and DUF336 are commonly present in *A. xylosoxidans* isolates and not specific to the type strain used in the LC-MS/MS experiment. PCR amplification of the genes corresponding to the three antigens was therefore performed in a collection of 14 *A*. *xylosoxidans* isolates. Among these isolates, five were obtained from the individuals in the CF_Achro_ serum group. All genes could be amplified in all isolates (Fig. S3), suggesting that the three genes are common across *A. xylosoxidans* and not unique to the type strain used in this study.

**Fig 4:**
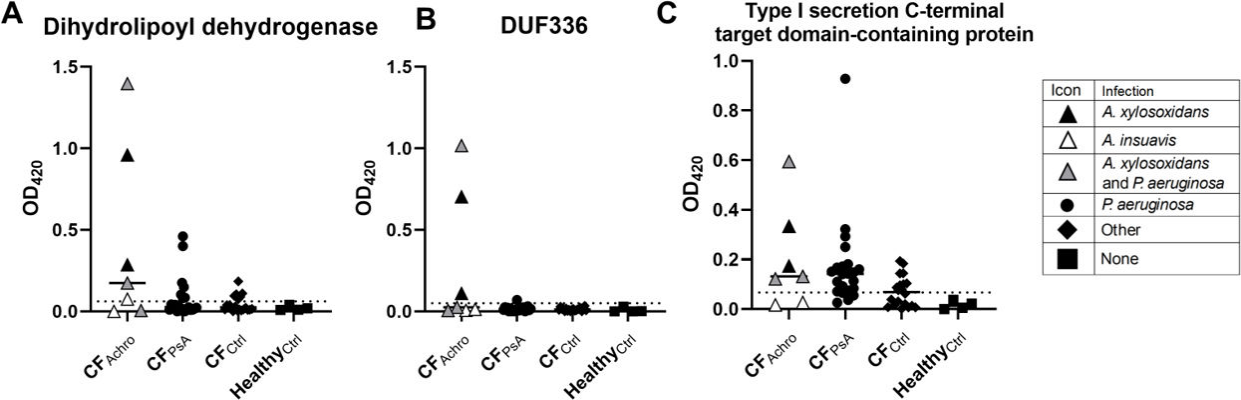
Serum IgG binding to recombinantly expressed *A. xylosoxidans* antigens. ELISA plates were coated with recombinantly expressed dihydrolipoyl dehydrogenase (**A**), domain of unknown function (DUF336) (**B**), and type I secretion C-terminal target domain-containing protein (**C**), and IgG titers in sera from pwCF and healthy controls were measured by direct ELISA. Each dot represents the average value of three technical replicates, and bars represent the mean value. The dotted lines represent the average of the healthy control group + 3 SD, which was considered the cutoff for positivity.

Having confirmed that all three antigens seem to be universally present in *A. xylosoxidans* isolates, we investigated the presence of specific IgG in a larger cohort of sera from pwCF. To this end, the recombinantly expressed antigens were used in an ELISA to quantify IgG titers in all 53 sera included in the study. As in the initial ELISA using whole bacteria, sera from two patients infected with *A. xylosoxidans* stood out as having high titers against all three antigens. Using the mean OD value +three standard deviations of the healthy control group as a cut-off for positivity, 71% of the CF_Achro_ sera had positive IgG titers against DLD and T1S-DCP, whereas 43% were positive against DUF336 (Fig. 4; Table 2). T1S-DCP was the least specific antigen, displaying a large proportion of positive serum titers in all groups except healthy controls (Fig. 4C; Table 2). Taken together, antibody responses to DUF336 and possibly also DLD appear to be specific to *Achromobacter* infection.

**TABLE 2:** Serum samples with positive titers against recombinant proteins

Positive samples	CF_Achro_ (*n* = 7)	CF_PsA_ (*n* = 22)	*P* value^*a*^ (CF_Achro_ vs CF_PsA_)	CF_Ctrl_ (*n* = 20)	*P* value^*a*^ (CF_Achro_ vs CF_Ctrl_)
DLD	5 (71%)	7 (32%)	0.06	6 (30%)	0.05
DUF336	3 (43%)	1 (5%)	0.01	0 (0%)	0.001
T1S-DCP	5 (71%)	18 (82%)	0.55	10 (50%)	0.33

^*a*^ Chi-square test.

## DISCUSSION

In the present study, we report that 57% (4/7) pwCF infected with *Achromobacter* spp. develop circulating IgG against whole bacteria, and we present an in-depth characterization of *Achromobacter* protein antigens that trigger an antibody host response. While some studies have attempted to discover lymphocyte epitopes using predictive tool approaches *in silico* (18, 19), our study is to our knowledge the first to investigate *A. xylosoxidans* antigens using patient serum IgG for affinity purification of bacterial proteins. Using his method, we identified eight antigens associated with CF_Achro_ serum-IgG, four of which (T1S-DCP, DLD, DUF336, and ABC-TSBP) did not show cross-reactivity with anti-*Pseudomonas* IgG. The role or function of these four antigens has not been previously characterized in *A. xylosoxidans*. However, T1S-DCP is associated with the type I secretion system, which mediates translocation of proteins across the cell membrane (20). The DUF336 was predicted by sequence homology to be part of a heme-binding protein (File S3), possibly as a hemophore (21). DLD exhibits dehydrogenase activity and has been implicated in complement evasion in *P. aeruginosa* (22, 23). Finally, ABC-TSBP is associated with ABC transporters, which have been implicated as therapeutic and vaccine targets as they are essential for the translocation of a wide variety of substrates across cell membranes (24).

Three of the antigens identified in the pull-down experiments (T1S-DCP, DLD, and DUF336) were expressed recombinantly, and antibody titers against recombinant proteins were confirmed in several CF_Achro_ sera. The corresponding genes were also confirmed to be present in all *Achromobacter* spp. isolates tested in this study. Taken together, these antigens could be potential candidates for serologic testing of *Achromobacter* serum antibodies. With the introduction of CFTR modulator therapies, the use of serological approaches in CF infection diagnostics may come to play a greater role (25). After the start of CFTR modulator treatment, many pwCF experience a marked reduction in sputum production and dramatically improved lung function and quality of life. However, pathogenic microorganisms that colonize the airways may remain or rebound despite modulator therapy (26, 27), and reduced sputum production can limit the possibility of monitoring chronic airway infections via surveillance cultures (28). Serological assays have been implicated for clinical routine use to monitor *P. aeruginosa* infection status (29), as serum antibodies to *P. aeruginosa* have the potential for early detection of chronic *P. aeruginosa* infection (30, 31).

However, the utility of serum antibodies against bacteria in pwCF may be limited by cross-reactivity between CF pathogens (32). A strength of the present study is the comparison of antigens retrieved from CF_Achro_ and CF_PsA_ serum-IgG, respectively, which allows the exclusion of cross-reactive antigens. Our data suggest that especially the *A. xylosoxidans* antigens DLD and DUF336 could be interesting candidates for the detection of specific *Achromobacter* serum IgG in serologic assays. However, the results need to be further validated against larger patient cohorts.

Although IgA is the dominating immunoglobulin in airway secretions, IgG is present in the lower respiratory tract and plays an important role in airway immunity. IgG in the airways comes from local secretion from the airway mucosa and intraluminal lymphocytes, or via transudation from plasma (33). In CF, titers of circulating IgG against specific pathogens have been associated with low lung function and a worse prognosis. For example, the presence of precipitating serum antibodies against *Achromobacter* has previously been demonstrated by Rønne Hansen et al. 11, who also described great variability between individuals and a correlation between higher precipitin levels and a more rapid lung function decline. Similarly, rapidly increasing *Pseudomonas* precipitin levels are associated with poor prognosis (34), suggesting that antibody titers reflect the severity of infection.

Notably, neither of the two patients infected with *A. insuavis* in our study had circulating IgG against *Achromobacter*, whereas four out of five patients with *A. xylosoxidans* infection had positive serum titers. *A. insuavis* exoproducts are less potent in triggering inflammatory responses from airway epithelial cells *in vitro* compared to *A. xylosoxidans* (14), and it could be speculated that these findings suggest a difference in pathogenicity and immune evasion patterns between the two species. However, larger studies on clinical outcomes would be necessary to determine such differences.

The most important limitation of this study is the low availability of sera from pwCF colonized with *Achromobacter*. Due to the limited number of *Achromobacter* cases, we cannot investigate any associations between antibody titers and duration of infection, lung function, or effects of exacerbations and antibiotic treatment. The data are further confounded by the presence of both *A. xylosoxidans* and *A. insuavis* infections in the *Achromobacter* cohort. As the infection is uncommon in an already relatively small patient group, multi-center studies would be required to increase the sample size. Moreover, only one CF_Achro_ serum sample was used for the identification of *A. xylosoxidans* antigens. Although IgG against the identified antigens could be verified in serum from other pwCF, additional relevant antigens may have been captured in other serum samples due to individual variations. There is also a possibility of false-negative results arising from the limited statistical power of the analysis. Additionally, we were only able to identify protein antigens expressed by the specific bacterial isolate under the growth conditions used in this study. Other relevant antigens may be expressed during *in vivo* growth in the CF lung. Moreover, the antigens investigated in this study were obtained from the secretome and surface proteome fractions. There may be additional IgG targets, such as cytosolic proteins or polysaccharide antigens, which were not included in this experimental setup. However, the identified protein antigens provided a similar pattern of IgG titers in patient serum when compared to serum titers against whole bacteria, suggesting that they are representative of a host antibody response against *Achromobacter* infection.

In conclusion, most pwCF with *A. xylosoxidans* airway infection raise an antibody response against *A. xylosoxidans*. We identified four specific *A. xylosoxidans* antigens, out of which three were expressed recombinantly and validated by ELISA. In particular, DUF336 and DLD were highly specific to the sera from pwCF with *A. xylosoxidans* infection and could be candidates for the development of a serological assay.

## Data Availability

The mass spectrometry data are openly available via the MassIVE repository (https://massive.ucsd.edu/) with the data set identifier MSV000095263.

## SUPPLEMENTAL MATERIAL

File S1 msphere.00233-25-s0001.xlsx Filtered MS results of bacterial protein contents.

File S2 msphere.00233-25-s0002.xlsx Filtered MS results of affinity purified antigenic candidates.

File S3 msphere.00233-25-s0003.pdf BLAST and CLUSTALW alignment results of candidate antigen homology.

Supplemental figures msphere.00233-25-s0004.pdf Figures S1 to S3.

Supplemental legends msphere.00233-25-s0005.pdf Supplemental file and figure legends.

ASM does not own the copyrights to Supplemental Material that may be linked to, or accessed through, an article. The authors have granted ASM a non-exclusive, world-wide license to publish the Supplemental Material files. Please contact the corresponding author directly for reuse.
