# Supplementary material for: Antibody-guided identification of Achromobacter xylosoxidans protein antigens in cystic fibrosis
Source: mSphere. 2025 Apr 29;10(5):e00233-25. doi: 10.1128/msphere.00233-25 (PMC12108089; doi:10.1128/msphere.00233-25)
Supplement: File S3 — BLAST and CLUSTALW alignment results of candidate antigen homology. [file msphere.00233-25-s0003.pdf]

>tr|A0A0D6GPL2|A0A0D6GPL2_ALCXX Dihydrolipoyl dehydrogenase
MSKQFDVVVIGAGPGGYIAAIRAAQLGMSVACIDAWQNGQGGPAPGGTCTNVGCIPSKAL
LQSSEHFEQANHHFAEHGIEVKGVSLKLDTLIGRKNSVVKQNNDGILYLFKKNKVTFFHG
KGAFAGQVEGGWAIKVTGTAEEDLVAKHVVVATGSSARELPGLPFDEKVVLSNDGALNIG
AVPKTLGVIGAGVIGLEMGSVWRRLGAEVTILEAMPEFLAAADQQVAKEALKAFTKQGLN
IQMGVKIGEIKATAKSVTVPYVDAKGAEQKLVVDKLIVSIGRVPYTGGLNADAVGLKLDE
RGFIAVDGDCKTNLPNVWAVGDVVRGPMLAHKAEEEGVAVAERIAGQHGHVNFDTVPWVI
YTSPEIAWVGKTEQQLKAEGREYKAGSFPFLANGRARALGDTTGFAKVIADAKTDEVLGV
HIVGPMASELISEAVTIMEFRGAAEDIARICHAHPTLSEAVKEAALAVDKRALNF

	Description	Scientific Name	Max Score	Total Score	Query Cover	E value	Per. Ident	Acc. Len	Accession
☑	dihydrolipoyl dehydrogenase [Achromobacter insuavis]	Achromobacter insuavis	296	296	99%	1e-95	37.03%	467	WP_241132288.1

dihydrolipoyl dehydrogenase [Achromobacter insuavis]

Sequence ID: WP_241132288.1 Length: 467 Number of Matches: 1

Range 1: 5 to 467 GenPept Graphics ▼ Next Match ▲ Previous Match

Score	Expect	Method	Identities	Positives	Gaps
296 bits(758)	1e-95	Compositional matrix adjust.	177/478(37%)	270/478(56%)	19/478(3%)

```
Query  2    SKQFDVVVIGAGPGGYIAAIRAQLGMSVACIDAWQNGQGGPAPGGTCTNVGCIPSKALL  61
            +K   ++VIG GPGGY+AAIRA QLG+    ++       G   GGTC N+GCIPSKAL+
Sbjct  5    TKTTTLLVIGGGPGGYVAAIRAGQLGVPTILVE-------GDRLGGTCLNIGCIPSKALI  57

Query  62   QSSEHFEQANHHFAEH--GIEVKGVSLKLDTLIGRKNSVVKQNNDGILYLFKKNKVTFFH  119
             ++E F++A H+  +   GI V   ++ +   +  K+ +V +   G+  L KKN V   H
Sbjct  58   HAAEEFDKARHYAGQSALGISVSAPAIDIGRTVAWKDGIVGKLTGGVGALLKKNGVQVVH  117

Query  120  GKGAFAGQVEGGWA-IKVTGTAEEDLVAKHVVVATGSSARELPGLPFDEKVVLSNDGALN  178
            G   +A  ++G    ++    + + +  +H+++A GS    LP +PF   VV S + AL+
Sbjct  118  G---WASLLDGKTVEVESADGSRQRIQCEHLLLAAGSEPTPLPSVPFGGMVVSSTE-ALS  173

Query  179  IGAVPKTLGVIGAGVIGLEMGSVWRRLGAEVTILEAMPEFLAAADQQVAKEALKAFTKQG  238
               +PK L V+G G IGLE+G+V+R+LGAEV ++EA    L   D ++ K    A  K G
Sbjct  174  PADIPKKLVVVGGGYIGLELGTVYRKLGAEVAVVEAQDRILPTYDAELTKPVAAALAKLG  233

Query  239  LNIQMGVKIGEIKATAKSVTVPYVDAKGAEQKLVVDKLIVSIGRVPYTGGLNADAVGLKL  298
            + + +G K+  +     +V +   DA GAE  L  D++++++GR P T G   +   L+L
Sbjct  234  VELHLGRKVLGLNGAGTAVRIQ--DASGAETALPADRVLIAVGRRPRTQGWGLE--NLQL  289

Query  299  DERG-FIAVDGDCKTNLPNVWAVGDVVRGPMLAHKAEEEGVAVAERIAGQHGHVNFDTVP  357
            D +G  + ++  C+T++ +VWA+GD+   PMLAH+A  +G  VAE +AG+  H     +P
Sbjct  290  DRKGNALRIEDQCRTSMRDVWAIGDIAGEPMLAHRAMAQGEMVAELVAGKRRHFQPAAIP  349

Query  358  WVIYTSPEIAWVGKTEQQLKAEGREYKAGSFPFLANGRARALGDTTGFAKVIADAKTDEV  417
             V +T PE+   G +  + +A G +  A SFPF ANGRA  L  T GF +V+A      +
Sbjct  350  AVCFTDPEVVVAGLSPAEAEAAGLDCLAASFPFAANGRAMTLESTDGFVRVVARRDNHLI  409

Query  418  LGVHIVGPMASELISEAVTIMEFRGAAEDIARICHAHPTLSEAVKEAALAVDKRALNF  475
            +G   VG   SEL +     +E     ED+A   HAHPTL EAV+EAAL     AL+
Sbjct  410  VGWQAVGRGVSELSTAFGQSLEMGATLEDVAGTIHAHPTLGEAVQEAALKALGHALHI  467
```

>WP_241132288.1 dihydrolipoyl dehydrogenase [Achromobacter insuavis]
MSQTTKTTTLLVIGGGPGGYVAAIRAGQLGVPTILVEGDRLGGTCLNIGCIPSKALIHAAEEFDKARHYA
GQSALGISVSAPAIDIGRTVAWKDGIVGKLTGGVGALLKKNGVQVVHGWASLLDGKTVEVESADGSRQR
IQCEHLLLAAGSEPTPLPSVPFGGMVVSSTEALSPADIPKKLVVVGGGYIGLELGTVYRKLGAEVAVVEAQ
DRILPTYDAELTKPVAAALAKLGVELHLGRKVLGLNGAGTAVRIQDASGAETALPADRVLIAVGRRPRTQG
WGLENLQLDRKGNALRIEDQCRTSMRDVWAIGDIAGEPMLAHRAMAQGEMVAELVAGKRRHFQPAAI
PAVCFTDPEVVVAGLSPAEAEAAGLDCLAASFPFAANGRAMTLESTDGFVRVVARRDNHLIVGWQAVGR
GVSELSTAFGQSLEMGATLEDVAGTIHAHPTLGEAVQEAALKALGHALHI

	Description	Scientific Name	Max Score	Total Score	Query Cover	E value	Per. Ident	Acc. Len	Accession
☑	dihydrolipoyl dehydrogenase [Pseudomonas aeruginosa]	Pseudomonas aer...	682	682	100%	0.0	68.00%	475	ELK4836048.1

dihydrolipoyl dehydrogenase [Pseudomonas aeruginosa]

Sequence ID: ELK4836048.1 Length: 475 Number of Matches: 1

See 1 more title(s) ⌄ See all Identical Proteins(IPG)

Range 1: 1 to 475 GenPept Graphics ▼ Next Match ▲ Previous Match

Score	Expect	Method	Identities	Positives	Gaps
682 bits(1761)	0.0	Compositional matrix adjust.	323/475(68%)	384/475(80%)	0/475(0%)

```
Query  1    MSKQFDVVVIGAGPGGYIAAIRAAQLGMSVACIDAWQNGQGGPAPGGTCTNVGCIPSKAL  60
            M   FDV+VIGAGPGGYIAAIRAAQLGM VAC+DAW+N  G PAPGGTC N+GCIPSKAL
Sbjct  1    MDMHFDVIVIGAGPGGYIAAIRAAQLGMKVACVDAWKNKDGKPAPGGTCNNIGCIPSKAL  60

Query  61   LQSSEHFEQANHHFAEHGIEVKGVSLKLDTLIGRKNSVVKQNNDGILYLFKKNKVTFFHG  120
            LQSSE+FEQA HHF  HGI    + + + T++ RKN VVK +N+GILYLF+KNKV FF+G
Sbjct  61   LQSSENFEQAKHHFGTHGISTGDLRMDVTTMLERKNQVVKSSNEGILYLFRKNKVQFFNG  120

Query  121  KGAFAGQVEGGWAIKVTGTAEEDLVAKHVVVATGSSARELPGLPFDEKVVLSNDGALNIG  180
              +F   V+GG+ + V       LV K ++VATGS+ R LP LPFDE+VVLSNDGAL+I
Sbjct  121  LASFTRTVDGGFEVSVAADEAVTLVGKQIIVATGSNVRPLPNLPFDERVVLSNDGALDIA  180

Query  181  AVPKTLGVIGAGVIGLEMGSVWRRLGAEVTILEAMPEFLAAADQQVAKEALKAFTKQGLN  240
            AVP  L VIGAGVIGLE+GSVWRRLGA+VTILE +P FL   DQ +AKEA KAF KQGL
Sbjct  181  AVPDRLAVIGAGVIGLELGSVWRRLGADVTILEGLPSFLPIVDQAIAKEAKKAFDKQGLK  240

Query  241  IQMGVKIGEIKATAKSVTVPYVDAKGAEQKLVVDKLIVSIGRVPYTGGLNADAVGLKLDE  300
            I++G K+ E+ AT   VT+ Y D++G  Q L  DK+IV+IGRVP T GLN  AVGL+LDE
Sbjct  241  IELGAKVREVNATEAGVTIHYTDSQGQTQSLQADKVIVAIGRVPNTEGLNPAAVGLQLDE  300

Query  301  RGFIAVDGDCKTNLPNVWAVGDVVRGPMLAHKAEEEGVAVAERIAGQHGHVNFDTVPWVI  360
            RG + VD +C+T++P +WA+GDVVRGPMLAHKAEEEGVAVAERIAGQHGHV+F+T+P VI
Sbjct  301  RGAVLVDDECRTSVPGIWAIGDVVRGPMLAHKAEEEGVAVAERIAGQHGHVDFNTIPNVI  360

Query  361  YTSPEIAWVGKTEQQLKAEGREYKAGSFPFLANGRARALGDTTGFAKVIADAKTDEVLGV  420
            YTSPEIAWVG+TEQQLK +G  Y+ GSFPF+ANGRARALGDTTG  KVIAD+ TDE+LGV
Sbjct  361  YTSPEIAWVGRTEQQLKEQGTAYRIGSFPFMANGRARALGDTTGLVKVIADSATDEILGV  420

Query  421  HIVGPMASELISEAVTIMEFRGAAEDIARICHAHPTLSEAVKEAALAVDKRALNF  475
            H+VGP ASEL++EAV  + F+ ++EDIARIC AHPTLSE  KEA+LAVDKRALNF
Sbjct  421  HVVGPQASELVAEAVIAIAFKASSEDIARICFAHPTLSETFKEASLAVDKRALNF  475
```

>ELK4836048.1 dihydrolipoyl dehydrogenase [Pseudomonas aeruginosa]

MDMHFDVIVIGAGPGGYIAAIRAAQLGMKVACVDAWKNKDGKPAPGGTCNNIGCIPSKALLQSSENFE
QAKHHFGTHGISTGDLRMDVTTMLERKNQVVKSSNEGILYLFRKNKVQFFNGLASFTRTVDGGFEVSVA
ADEAVTLVGKQIIVATGSNVRPLPNLPFDERVVLSNDGALDIAAVPDRLAVIGAGVIGLELGSVWRRLGA
DVTILEGLPSFLPIVDQAIAKEAKKAFDKQGLKIELGAKVREVNATEAGVTIHYTDSQGQTQSLQADKVIVA
IGRVPNTEGLNPAAVGLQLDERGAVLVDDECRTSVPGIWAIGDVVRGPMLAHKAEEEGVAVAERIAGQ
HGHVDFNTIPNVIYTSPEIAWVGRTEQQLKEQGTAYRIGSFPFMANGRARALGDTTGLVKVIADSATDEIL
GVHVVGPQASELVAEAVIAIAFKASSEDIARICFAHPTLSETFKEASLAVDKRALNF

```
 CLUSTAL 2.1 Multiple Sequence Alignments


Sequence type explicitly set to Protein
Sequence format is Pearson
Sequence 1: tr|A0A0D6GPL2|A0A0D6GPL2_ALCXX   475 aa
Sequence 2: WP_241132288.1                   467 aa
Sequence 3: ELK4836048.1                     475 aa
Start of Pairwise alignments
Aligning...


Sequences (1:2) Aligned. Score: 31.9058
Sequences (1:3) Aligned. Score: 68
Sequences (2:3) Aligned. Score: 35.3319
Guide tree file created:   [clustalw.dnd]

There are 2 groups
Start of Multiple Alignment

Aligning...
Group 1: Sequences:   2      Score:7238
Group 2: Sequences:   3      Score:3239
Alignment Score 4057

CLUSTAL-Alignment file created  [clustalw.aln]
```

---

clustalw.aln

```
CLUSTAL 2.1 multiple sequence alignment


tr|A0A0D6GPL2|A0A0D6GPL2_ALCXX      ---MSKQFDVVVIGAGPGGYIAAIRAAQLGMSVACIDAWQNGQGGPAPGG
ELK4836048.1                        ---MDMHFDVIVIGAGPGGYIAAIRAAQLGMKVACVDAWKNKDGKPAPGG
WP_241132288.1                      MSQTTKTTTLLVIGGGPGGYVAAIRAGQLGVPTILVEGDR-------LGG
                                             ::***.*****:*****.***: .  ::. :        **

tr|A0A0D6GPL2|A0A0D6GPL2_ALCXX      TCTNVGCIPSKALLQSSEHFEQANHHFAE--HGIEVKGVSLKLDTLIGRK
ELK4836048.1                        TCNNIGCIPSKALLQSSENFEQAKHHFGT--HGISTGDLRMDVTTMLERK
WP_241132288.1                      TCLNIGCIPSKALIHAAEEFDKARHYAGQSALGISVSAPAIDIGRTVAWK
                                    ** *:********::::*.*::*.*: .    **..    :.:   :  *

tr|A0A0D6GPL2|A0A0D6GPL2_ALCXX      NSVVKQNNDGILYLFKKNKVTFFHGKGAFAGQVEGGWAIKVTGTAEEDLV
ELK4836048.1                        NQVVKSSNEGILYLFRKNKVQFFNGLASFTRTVDGGFEVSVAADEAVTLV
WP_241132288.1                      DGIVGKLTGGVGALLKKNGVQVVHGWASLLDGKT--VEVESADGSRQRIQ
                                    : :* . . *:  *::** * ..:* .::         :. :      :

tr|A0A0D6GPL2|A0A0D6GPL2_ALCXX      AKHVVVATGSSARELPGLPFDEKVVLSNDGALNIGAVPKTLGVIGAGVIG
ELK4836048.1                        GKQIIVATGSNVRPLPNLPFDERVVLSNDGALDIAAVPDRLAVIGAGVIG
WP_241132288.1                      CEHLLLAAGSEPTPLPSVPFGG-MVVSSTEALSPADIPKKLVVVGGGYIG
                                     :::::*:**.   **.:**.  :*:*.  **. . :*. * *:*.* **

tr|A0A0D6GPL2|A0A0D6GPL2_ALCXX      LEMGSVWRRLGAEVTILEAMPEFLAAADQQVAKEALKAFTKQGLNIQMGV
ELK4836048.1                        LELGSVWRRLGADVTILEGLPSFLPIVDQAIAKEAKKAFDKQGLKIELGA
WP_241132288.1                      LELGTVYRKLGAEVAVVEAQDRILPTYDAELTKPVAAALAKLGVELHLGR
                                    **:*:*:*:***:*:::*.   :*.  *  ::* .  *: * *:::.:*

tr|A0A0D6GPL2|A0A0D6GPL2_ALCXX      KIGEIKATAKSVTVPYVDAKGAEQKLVVDKLIVSIGRVPYTGGLNADAVG
ELK4836048.1                        KVREVNATEAGVTIHYTDSQGQTQSLQADKVIVAIGRVPNTEGLNPAAVG
WP_241132288.1                      KVLGLNG--AGTAVRIQDASGAETALPADRVLIAVGRRPRTQGWG--LEN
                                    *:  ::.   ..::   *:.*    * .*::::::** * * * .    .

tr|A0A0D6GPL2|A0A0D6GPL2_ALCXX      LKLDERG-FIAVDGDCKTNLPNVWAVGDVVRGPMLAHKAEEEGVAVAERI
ELK4836048.1                        LQLDERG-AVLVDDECRTSVPGIWAIGDVVRGPMLAHKAEEEGVAVAERI
WP_241132288.1                      LQLDRKGNALRIEDQCRTSMRDVWAIGDIAGEPMLAHRAMAQGEMVAELV
                                    *:**.:*  : ::.:*:*.: .:**:**:.  *****:*  :*  *** :

tr|A0A0D6GPL2|A0A0D6GPL2_ALCXX      AGQHGHVNFDTVPWVIYTSPEIAWVGKTEQQLKAEGREYKAGSFPFLANG
ELK4836048.1                        AGQHGHVDFNTIPNVIYTSPEIAWVGRTEQQLKEQGTAYRIGSFPFMANG
WP_241132288.1                      AGKRRHFQPAAIPAVCFTDPEVVVAGLSPAEAEAAGLDCLAASFPFAANG
                                    **:: *.:  ::* * :*.**:. .* :  : :  *     .**** ***

tr|A0A0D6GPL2|A0A0D6GPL2_ALCXX      RARALGDTTGFAKVIADAKTDEVLGVHIVGPMASELISEAVTIMEFRGAA
ELK4836048.1                        RARALGDTTGLVKVIADSATDEILGVHVVGPQASELVAEAVIAIAFKASS
WP_241132288.1                      RAMTLESTDGFVRVVARRDNHLIVGWQAVGRGVSELSTAFGQSLEMGATL
                                    ** :* .* *:.:*:*   .. ::* : **  .*** :     : : .:

tr|A0A0D6GPL2|A0A0D6GPL2_ALCXX      EDIARICHAHPTLSEAVKEAALAVDKRALNF
ELK4836048.1                        EDIARICFAHPTLSETFKEASLAVDKRALNF
WP_241132288.1                      EDVAGTIHAHPTLGEAVQEAALKALGHALHI
                                    **:*   .*****.*:.:**:* .  :**::
```

>tr|A0A0D6GMC1|A0A0D6GMC1_ALCXX Domain of uncharacterized function (DUF336)
MNTKPVLTAEDVKKILAAAEAHALQNKWAVTIAVSDDGGHLLGMLRLDDAAPISSHIAPA
KAKTAALGRRESRVYEEIINNGRYSFLSAPLIEGMLEGGVPIVANGQVVGAVGVSGVKST
EDAQIAQAGIAALGL

	Description	Scientific Name	Max Score	Total Score	Query Cover	E value	Per. Ident	Acc. Len	Accession
☑	heme-binding protein [Achromobacter insuavis]	Achromobacter insuavis	262	262	100%	3e-92	97.04%	135	WP_241070600.1

heme-binding protein [Achromobacter insuavis]

Sequence ID: WP_241070600.1 **Length:** 135 **Number of Matches:** 1

Range 1: 1 to 135 GenPept Graphics ▼ Next Match ▲ Previous Match

Score	Expect	Method	Identities	Positives	Gaps
262 bits(670)	3e-92	Compositional matrix adjust.	131/135(97%)	132/135(97%)	0/135(0%)

```
Query  1    MNTKPVLTAEDVKKILAAAEAHALQNKWAVTIAVSDDGGHLLGMLRLDDAAPISSHIAPA  60
            MNTKPVL AEDVKKILAAAEAHALQNKWAVTIAVSDDGGHLLGMLRLDDAAPISSHIAPA
Sbjct  1    MNTKPVLNAEDVKKILAAAEAHALQNKWAVTIAVSDDGGHLLGMLRLDDAAPISSHIAPA  60

Query  61   KAKTAALGRRESRVYEEIINNGRYSFLSAPLIEGMLEGGVPIVANGQVVGAVGVSGVKST  120
            KAKTAALGRRESRVYEEIINNGRYSFLSAPLIEGMLEGGVPI  NGQVVGAVGVSGVKST
Sbjct  61   KAKTAALGRRESRVYEEIINNGRYSFLSAPLIEGMLEGGVPITVNGQVVGAVGVSGVKST  120

Query  121  EDAQIAQAGIAALGL  135
            EDAQ+AQAGIAALGL
Sbjct  121  EDAQVAQAGIAALGL  135
```

>WP_241070600.1 heme-binding protein [Achromobacter insuavis]
MNTKPVLNAEDVKKILAAAEAHALQNKWAVTIAVSDDGGHLLGMLRLDDAAPISSHIAPAKAKTAALGR
RESRVYEEIINNGRYSFLSAPLIEGMLEGGVPITVNGQVVGAVGVSGVKSTEDAQVAQAGIAALGL

	Description	Scientific Name	Max Score	Total Score	Query Cover	E value	Per. Ident	Acc. Len	Accession
☑	heme-binding protein [Pseudomonas aeruginosa]	Pseudomonas aeruginosa	132	132	98%	7e-39	58.65%	132	WP_058201157.1

heme-binding protein [Pseudomonas aeruginosa]

Sequence ID: WP_058201157.1 Length: 132 Number of Matches: 1

See 5 more title(s) ˅ See all Identical Proteins(IPG)

Range 1: 1 to 132 GenPept Graphics ▼ Next Match ▲ Previous Match

Score	Expect	Method	Identities	Positives	Gaps
132 bits(333)	7e-39	Compositional matrix adjust.	78/133(59%)	107/133(80%)	1/133(0%)

```
Query  1    MNTKPVLTAEDVKKILAAAEAHALQNKWAVTIAVSDDGGHLLGMLRLDDAAPISSHIAPA  60
            M+ K VLT  +V +ILAAA A A +N+WAV IA+ DDGGH L + RLD  AP+ ++IA
Sbjct  1    MHHKAVLTQTEVARILAAARAEAQRNQWAVAIALVDDGGHPLALERLDGCAPVGAYIATE  60

Query  61   KAKTAALGRRESRVYEEIINNGRYSFLSAPLIEGMLEGGVPIVANGQVVGAVGVSGVKST  120
            KA+++ALGRRE++ YEE++N GR +F+SAPL+   LEGGVP++ +G+VVGAVGVSGVK+
Sbjct  61   KARSSALGRRETKGYEEMVNGGRTAFVSAPLLTS-LEGGVPVLVDGEVVGAVGVSGVKAE  119

Query  121  EDAQIAQAGIAAL  133
            +DAQ+A+AG+AAL
Sbjct  120  QDAQVAKAGVAAL  132
```

>WP_058201157.1 heme-binding protein [Pseudomonas aeruginosa]
MHHKAVLTQTEVARILAAARAEAQRNQWAVAIALVDDGGHPLALERLDGCAPVGAYIATEKARSSALGRRETKGYEEMVNGGRTAFVSAPLLTSLEGGVPVLVDGEVVGAVGVSGVKAEQDAQVAKAGVAAL

```
 CLUSTAL 2.1 Multiple Sequence Alignments


Sequence type explicitly set to Protein
Sequence format is Pearson
Sequence 1: tr|A0A0D6GMC1|A0A0D6GMC1_ALCXX   135 aa
Sequence 2: WP_241070600.1                   135 aa
Sequence 3: WP_058201157.1                   132 aa
Start of Pairwise alignments
Aligning...



Sequences (1:2) Aligned. Score: 97.037
Sequences (1:3) Aligned. Score: 56.8182
Sequences (2:3) Aligned. Score: 57.5758
Guide tree file created:   [clustalw.dnd]

There are 2 groups
Start of Multiple Alignment

Aligning...
Group 1: Sequences:   2      Score:2146
Group 2: Sequences:   3      Score:1132
Alignment Score 1721

CLUSTAL-Alignment file created  [clustalw.aln]
```

```
clustalw.aln

CLUSTAL 2.1 multiple sequence alignment


tr|A0A0D6GMC1|A0A0D6GMC1_ALCXX      MNTKPVLTAEDVKKILAAAEAHALQNKWAVTIAVSDDGGHLLGMLRLDDA
WP_241070600.1                      MNTKPVLNAEDVKKILAAAEAHALQNKWAVTIAVSDDGGHLLGMLRLDDA
WP_058201157.1                      MHHKAVLTQTEVARILAAARAEAQRNQWAVAIALVDDGGHPLALERLDGC
                                    *: *.**.  :* :*****.*.* :*:***:**: ***** *.: ***..

tr|A0A0D6GMC1|A0A0D6GMC1_ALCXX      APISSHIAPAKAKTAALGRRESRVYEEIINNGRYSFLSAPLIEGMLEGGV
WP_241070600.1                      APISSHIAPAKAKTAALGRRESRVYEEIINNGRYSFLSAPLIEGMLEGGV
WP_058201157.1                      APVGAYIATEKARSSALGRRETKGYEEMVNGGRTAFVSAPLLT-SLEGGV
                                    **:.::**. **:::******:: ***::*.** :*:****:   *****

tr|A0A0D6GMC1|A0A0D6GMC1_ALCXX      PIVANGQVVGAVGVSGVKSTEDAQIAQAGIAALGL
WP_241070600.1                      PITVNGQVVGAVGVSGVKSTEDAQVAQAGIAALGL
WP_058201157.1                      PVLVDGEVVGAVGVSGVKAEQDAQVAKAGVAAL--
                                    *: .:*:***********: :***:*:**:***
```

>tr|A0A7T2RJE8 Type I secretion C-terminal target domain-containing protein
MKDAVDKFKGLSGKSTVHAIGIGTGVNEAYLKFFDNSSTTGTGTVRIEGTNITGAVGQPQ
IVNTAKDLAAALQGGSSSTDPAAVGNDIINGGAGHDIIFGDTLNTDGNVLNWASVGGRPA
DLVQGSGLKALQVFLEMRDGHAPTNGDLYQYIKDHHADFNLADDPRGGDDTIHGGTGDDI
IYGQGGNDTLYGDDGNDIIYGGAGDDKLYGGEGNDVLHGGSGNDTLEGGNGNDLLIGGKG
DDTLIGGAGSDTFKWELGDQGTTAKPAVDTIKDFSLDKPADGGDVLDLKDLLVGEKDGTL
TQYLNFHKEGNNTVIDVNTQGKLGTQGADQKIVLENVDLTQGGQLNNQAIINDLLQKGKL
NVDHS

	Description	Scientific Name	Max Score	Total Score	Query Cover	E value	Per. Ident	Acc. Len	Accession
☑	calcium-binding protein [Achromobacter insuavis]	Achromobacter insuavis	62.4	471	26%	2e-10	59.26%	1039	WZB76692.1

calcium-binding protein [Achromobacter insuavis]

Sequence ID: WZB76692.1 **Length:** 1039 **Number of Matches:** 9

Range 1: 958 to 1011 GenPept Graphics ▼ Next Match ▲ Previous Match

Score	Expect	Method	Identities	Positives	Gaps
62.4 bits(150)	2e-10	Compositional matrix adjust.	32/54(59%)	39/54(72%)	0/54(0%)

```
Query  168   GDDTIHGGTGDDIIYGQGGNDTLYGDDGNDIIYGGAGDDKLYGGEGNDVLHGGS  221
             GDD + GG GDD +YG GG+D LYG DG+D +YGG G D LYGG G + L GG+
Sbjct  958   GDDVLIGGAGDDTLYGHGGDDKLYGGDGDDTLYGGDGIDHLYGGAGRNTLVGGA  1011
```

Range 2: 953 to 1010 GenPept Graphics ▼ Next Match ▲ Previous Match ⩓ First Match

Score	Expect	Method	Identities	Positives	Gaps
62.4 bits(150)	2e-10	Compositional matrix adjust.	33/58(57%)	43/58(74%)	0/58(0%)

```
Query  172   IHGGTGDDIIYGQGGNDTLYGDDGNDIIYGGAGDDKLYGGEGNDVLHGGSGNDTLEGG  229
             + G  GDD++ G  G+DTLYG  G+D +YGG GDD LYGG+G D L+GG+G +TL GG
Sbjct  953   LEGTAGDDVLIGGAGDDTLYGHGGDDKLYGGDGDDTLYGGDGIDHLYGGAGRNTLVGG  1010
```

Range 3: 953 to 1011 GenPept Graphics ▼ Next Match ▲ Previous Match ⩓ First Match

Score	Expect	Method	Identities	Positives	Gaps
61.2 bits(147)	5e-10	Compositional matrix adjust.	35/59(59%)	44/59(74%)	0/59(0%)

```
Query  190   LYGDDGNDIIYGGAGDDKLYGGEGNDVLHGGSGNDTLEGGNGNDLLIGGKGDDTLIGGA  248
             L G  G+D++ GGAGDD LYG  G+D L+GG G+DTL GG+G D L GG G +TL+GGA
Sbjct  953   LEGTAGDDVLIGGAGDDTLYGHGGDDKLYGGDGDDTLYGGDGIDHLYGGAGRNTLVGGA  1011
```

Range 4: 955 to 1011 GenPept Graphics ▼ Next Match ▲ Previous Match ⩓ First Match

Score	Expect	Method	Identities	Positives	Gaps
56.6 bits(135)	1e-08	Compositional matrix adjust.	31/57(54%)	39/57(68%)	0/57(0%)

```
Query  183   GQGGNDTLYGDDGNDIIYGGAGDDKLYGGEGNDVLHGGSGNDTLEGGNGNDLLIGGK  239
             G  G+D L G  G+D +YG  GDDKLYGG+G+D L+GG G D L GG G + L+GG 
Sbjct  955   GTAGDDVLIGGAGDDTLYGHGGDDKLYGGDGDDTLYGGDGIDHLYGGAGRNTLVGGA  1011
```

Range 5: 702 to 829 GenPept Graphics ▼ Next Match ▲ Previous Match ⩓ First Match

Score	Expect	Method	Identities	Positives	Gaps
50.4 bits(119)	1e-06	Compositional matrix adjust.	42/128(33%)	56/128(43%)	52/128(40%)

```
Query  183  GQGGNDTLYGDDGNDIIYGGAGDDKLYGGEGNDVLHGGSGNDT-----------------  225
            G   +D L GD  N++I GGAG+D++ GG GN+ L GG G DT
Sbjct  702  GSSYDDVLSGDSQNNVILGGAGNDRIAGGAGNNTLDGGVGIDTVDYSGAGAGVVVDLAAG  761

Query  226  -----------------------------------LEGGNGNDLLIGGKGDDTLIGGAGS  250
                                               L+GG GND+L G  G+D LIGGAG+
Sbjct  762  SAQNGLGGVDTLSNFENVTGSAYADKLSGNALDNVLDGGRGNDILDGRGGNDVLIGGAGN  821

Query  251  DTFKWELG  258
            DT+ ++LG
Sbjct  822  DTYLFDLG  829
```

>WZB76692.1 calcium-binding protein [Achromobacter insuavis]
MLFDGLVHANELIASNYGDTLIGGAWQTVLRGGAGHDTLVVTGTGYYMDGGGGSDTVSYAQWQRGVS
VSLARDSDDLGSTLAGIENLIGTGWADRLTGNGGGNRLDGGAGDDVLVGGGGNDVYVFGRGYGADTV
QNGIAANVGASSMIRVSAGIGIGDLWYERRGDDLLIRILGTKDALAVQGWYQEAFRKVAILELQGGLRLD
AAAIESLVESMQAWRQANPGFDPAVGQPRPPLDNIAPHYRNDYELPVVGEPVDVALETRQLLNSGKIAN
ALADVRAVAGSMASDRNNLNNYLATANQHGAAVTPIFIPEGWRLYRYTSSLEAGELITVSRFDWSNPNPL
LGRPSDVTSYVQLSGAEAGRFYYNVRTTSNMTGYGTRDSLKEGPAGQVGALLGYGNQVMGSMNQLSAY
AASYDSAFTARQSALNAAVAANTAATATSAAAATQAAQQFSAQLWTALYNYQAYGATLAGMQSQLAAYR
QYLAGAEPPAKYRKTADSFWMDYETSFYSSTDSARYQAFLALWQQGTNAYNNAVRFAGSFVNSLRGLD
NFQQAHYAAGGQTVQAGAGGDLLIAGSGANRRLVGGAGRDVFLFAQAAAGTVDDVLGFATGLSADRIW
LLQPSGDSAYVTVGPNGVILSYATAGGQAAQLRLNGVTLADLSLYDNLLGVRTVDFSRMGAGVSIKLDSL
TTRAADGYLHTSNLTGSSYDDVLSGDSQNNVILGGAGNDRIAGGAGNNTLDGGVGIDTVDYSGAGAGV
VVDLAAGSAQNGLGGVDTLSNFENVTGSAYADKLSGNALDNVLDGGRGNDILDGRGGNDVLIGGAGN
DTYLFDLGYGADRIVENDLTAGNTDTVLFGAGVRVQQLWFSRAGDDLVVAMPGTADRLTVTNWFLGAQY
QVEIFQTASGALLHAAAVQALVDAMAALSPTVPLAALTAQQQSALWPAVKTAWGLTRPEAIRLEGTAGDD
VLIGGAGDDTLYGHGGDDKLYGGDGDDTLYGGDGIDHLYGGAGRNTLVGGAATTSTMSTAPTTSSSSCL
TRVWTGYSLP

	Description	Scientific Name	Max Score	Total Score	Query Cover	E value	Per. Ident	Acc. Len	Accession
☑	calcium-binding protein [Pseudomonas aeruginosa]	Pseudomonas aeruginosa	98.2	231	41%	2e-21	44.44%	264	MDP5960240.1

calcium-binding protein [Pseudomonas aeruginosa]

Sequence ID: MDP5960240.1 Length: 264 Number of Matches: 3

Range 1: 51 to 191 GenPept Graphics ▼ Next Match ▲ Previous Match

Score	Expect	Method	Identities	Positives	Gaps
98.2 bits(243)	2e-21	Compositional matrix adjust.	68/153(44%)	88/153(57%)	17/153(11%)

```
Query  170  DTIHGGTGDDIIYGQGGNDTLYGDDGNDIIYGGAGDDKLYGGEGNDVLHGGSGNDTLEGG  229
            DT+ GG G+D +YG  GND L GD+GND +YGGAGDD L GG  +D L+G  G+D +EG
Sbjct  51   DTLSGGLGNDSLYGYAGNDLLQGDEGNDTLYGGAGDDTLIGGADSDYLYGEDGDDRIEGN  110

Query  230  NGNDLLIGGKGDDTLIGGAGSDTFKWELGDQGTTAKP-----AVDTIKDFSLDKPADGGD  284
            NGND L GG G+DTLIGG+G+D      GD G            DTI ++  +  A   D
Sbjct  111  NGNDTLYGGAGEDTLIGGSGNDYLA---GDAGNDIYQLGNGWGQDTINNYHTESNA--LD  165

Query  285  VLDLKDLLVGEKDGTLTQYLNFHKEGNNTVIDV  317
             L+  D +  +K       L F K GNN  I++
Sbjct  166  RLEFTDNITADK-------LWFSKNGNNLEINL  191
```

Range 2: 76 to 154 GenPept Graphics ▼ Next Match ▲ Previous Match ▲ First Match

Score	Expect	Method	Identities	Positives	Gaps
68.6 bits(166)	5e-11	Compositional matrix adjust.	43/79(54%)	53/79(67%)	2/79(2%)

```
Query  168  GDDTIHGGTGDDIIYGQGGNDTLYGDDGNDIIYGGAGDDKLYGGEGNDVLHGGSGNDTLE  227
            G+DT++GG GDD + G   +D LYG+DG+D I G  G+D LYGG G D L GGSGND L
Sbjct  76   GNDTLYGGAGDDTLIGGADSDYLYGEDGDDRIEGNNGNDTLYGGAGEDTLIGGSGNDYLA  135

Query  228  GGNGNDL--LIGGKGDDTL  244
            G  GND+  L  G G DT+
Sbjct  136  GDAGNDIYQLGNGWGQDTI  154
```

Range 3: 39 to 116 GenPept Graphics ▼ Next Match ▲ Previous Match ▲ First Match

Score	Expect	Method	Identities	Positives	Gaps
65.1 bits(157)	8e-10	Compositional matrix adjust.	42/78(54%)	48/78(61%)	0/78(0%)

```
Query  194  DGNDIIYGGAGDDKLYGGEGNDVLHGGSGNDTLEGGNGNDLLIGGKGDDTLIGGAGSDTF  253
            DGND ++G A  D L GG GND L+G +GND L+G  GND L GG GDDTLIGGA SD
Sbjct  39   DGNDRLFGYAVADTLSGGLGNDSLYGYAGNDLLQGDEGNDTLYGGAGDDTLIGGADSDYL  98

Query  254  KWELGDQGTTAKPAVDTI  271
              E GD         DT+
Sbjct  99   YGEDGDDRIEGNNGNDTL  116
```

>MDP5960240.1 calcium-binding protein, partial [Pseudomonas aeruginosa]
SSYFSQDATSNYRLEEIRFVDGQVLNIDTVKSLVQQATDGNDRLFGYAVADTLSGGLGNDSLYGYAGNDL
LQGDEGNDTLYGGAGDDTLIGGADSDYLYGEDGDDRIEGNNGNDTLYGGAGEDTLIGGSGNDYLAGD
AGNDIYQLGNGWGQDTINNYHTESNALDRLEFTDNITADKLWFSKNGNNLEINLIGASDKVSISNWYSG
KNYQISQFTAADGKTLLESQVQNLVNAMSSFGVPAGGESEMTVEQRQQLEVIIAANWQ

```
 CLUSTAL 2.1 Multiple Sequence Alignments


Sequence type explicitly set to Protein
Sequence format is Pearson
Sequence 1: tr|A0A7T2RJE8   365 aa
Sequence 2: WZB76692.1     1039 aa
Sequence 3: MDP5960240.1    264 aa
Start of Pairwise alignments
Aligning...



Sequences (1:2) Aligned. Score: 17.8082
Sequences (1:3) Aligned. Score: 25
Sequences (2:3) Aligned. Score: 26.1364
Guide tree file created:   [clustalw.dnd]

There are 2 groups
Start of Multiple Alignment

Aligning...
Group 1:                     Delayed
Group 2:                     Delayed
Alignment Score 815

CLUSTAL-Alignment file created  [clustalw.aln]
```

---

clustalw.aln

```
CLUSTAL 2.1 multiple sequence alignment


WZB76692.1          MLFDGLVHANELIASNYGDTLIGGAWQTVLRGGAGHDTLVVTGTGYYMDGGGGSDTVSYA
MDP5960240.1        ------------------------------------------------------------
tr|A0A7T2RJE8       ------------------------------------------------------------


WZB76692.1          QWQRGVSVSLARDSDDLGSTLAGIENLIGTGWADRLTGNGGGNRLDGGAGDDVLVGGGGN
MDP5960240.1        ------------------------------------------------------------
tr|A0A7T2RJE8       ------------------------------------------------------------


WZB76692.1          DVYVFGRGYGADTVQNGIAANVGASSMIRVSAGIGIGDLWYERRGDDLLIRILGTKDALA
MDP5960240.1        ------------------------------------------------------------
tr|A0A7T2RJE8       ------------------------------------------------------------


WZB76692.1          VQGWYQEAFRKVAILELQGGLRLDAAAIESLVESMQAWRQANPGFDPAVGQPRPPLDNIA
MDP5960240.1        ------------------------------------------------------------
tr|A0A7T2RJE8       ------------------------------------------------------------


WZB76692.1          PHYRNDYELPVVGEPVDVALETRQLLNSGKIANALADVRAVAGSMASDRNNLNNYLATAN
MDP5960240.1        ------------------------------------------------------------
tr|A0A7T2RJE8       ------------------------------------------------------------


WZB76692.1          QHGAAVTPIFIPEGWRLYRYTSSLEAGELITVSRFDWSNPNPLLGRPSDVTSYVQLSGAE
MDP5960240.1        ------------------------------------------------------------
tr|A0A7T2RJE8       -----------------------------------MKDAVDKFKGLSGKSTVHAIGIGTG


WZB76692.1          AGRFYYNVRTTSNMTGYGTRDSLKEGPAGQVGALLGYGNQVMGSMNQLSAYAASYDSAFT
MDP5960240.1        ------------------------------------------------------------
tr|A0A7T2RJE8       VNEAYLKFFDNSSTTGTGTVRIEGTNITGAVGQPQ-------------------------


WZB76692.1          ARQSALNAAVAANTAATATSAAAATQAAQQFSAQLWTALYNYQAYGATLAGMQSQLAAYR
MDP5960240.1        ------------------------------------------------------------
tr|A0A7T2RJE8       ------------------------------------------------------------


WZB76692.1          QYLAGAEPPAKYRKTADSFWMDYETSFYSSTDSARYQAFLALWQQGTNAYNNAVRFAGSF
MDP5960240.1        ------------------------------------------------------------
tr|A0A7T2RJE8       ------------------------------------------------------------
```

```
WZB76692.1         VNSLRGLDNFQQAHYAAGGQTVQAGAGGDLLIAGSGANRRLVGGAGRDVFLFAQAAAGTV
MDP5960240.1       ------------------------------------------------------------
tr|A0A7T2RJE8      --IVNTAKDLAAALQGGSSSTDPAAVGNDIINGGAGHDIIFG------------------


WZB76692.1         DDVLGFATGLSADRIWLLQPSGDSAYVTVGPNGVILSYATAGGQAAQLRLNGVTLADLSL
MDP5960240.1       ------------------------------------SSYFSQDATSNYRLEEIRFVDGQV
tr|A0A7T2RJE8      --------------------------DTLNTDGNVLNWASVGGRPADLVQGSGLKALQVF
                                                       .     . .::        .   .

WZB76692.1         YDNLLGVRTVDFSRMGAGVSIKLDSLTTRAADGYLHTSNLTGSSYDDVLSGDSQNNVILG
MDP5960240.1       LN--------------------IDTVKSLVQQATDGNDRLFGYAVADTLSGGLGNDSLYG
tr|A0A7T2RJE8      LEMRDG-----------HAPTNGDLYQYIKDHHADFNLADDPRGGDDTIHGGTGDDIIYG
                    :                     *       .    .      .  *.: *.  :: : *

WZB76692.1         GAGNDRIAGGAGNNTLDGGVGIDTVDYSGAGAGVVVDLAAGSAQNGLGGVDTLSNFENVT
MDP5960240.1       YAGNDLLQGDEGNDTLYGGAGDDTLIGG-------------------------ADSDYLY
tr|A0A7T2RJE8      QGGNDTLYGDDGNDIIYGGAGDD----------------------------------KLY
                    .*** : *. **: : **.* *                                   :

WZB76692.1         GSAYADKLSGNALDNVLDGGRGNDILDGRGGNDVLIGGAGNDTYLFDLGYGADRIVENDL
MDP5960240.1       GEDGDDRIEGNNGNDTLYGGAGEDTLIGGSGNDYLAGDAGNDIYQLGNGWGQDTINNYHT
tr|A0A7T2RJE8      GGEGNDVLHGGSGNDTLEGGNGNDLLIGGKGDDTLIGGAGSDTFKWELGD------QGTT
                   *    * : *.  ::.* ** *:* * *  *:* * *.**.* :    *       :

WZB76692.1         TAGNTDTVLFGAGVRVQQLWFSRAGDDLVVAMPGTADRLTVTNWFLGAQYQVEIFQTASG
MDP5960240.1       ESNALDRLEFTDNITADKLWFSKNGNNLEINLIGASDKVSISNWYSGKNYQISQFTAADG
tr|A0A7T2RJE8      AKPAVDTIKDFSLDKPADGGDVLDLKDLLVGEKDGTLTQYLNFHKEGNNTVIDVNTQGKL
                        * :         .       .:* :   . :    :.    * :  :.    ..

WZB76692.1         ALLHAAAVQALVDAMAAL-SPTVPLAALTAQQQSALWPAVKTAWGLTRPEAIRLEGTAGD
MDP5960240.1       KTLLESQVQNLVNAMSSFGVPAGGESEMTVEQRQQLEVIIAANWQ---------------
tr|A0A7T2RJE8      GTQGADQKIVLENVDLTQGGQLNNQAIINDLLQKGKLNVDHS------------------
                             * :.  :        : :.   :.       :

WZB76692.1         DVLIGGAGDDTLYGHGGDDKLYGGDGDDTLYGGDGIDHLYGGAGRNTLVGGAATTSTMST
MDP5960240.1       ------------------------------------------------------------
tr|A0A7T2RJE8      ------------------------------------------------------------


WZB76692.1         APTTSSSSCLTRVWTGYSLP
MDP5960240.1       --------------------
tr|A0A7T2RJE8      --------------------
```
