# Supplementary material for: Antibody-guided identification of Achromobacter xylosoxidans protein antigens in cystic fibrosis
Source: mSphere. 2025 Apr 29;10(5):e00233-25. doi: 10.1128/msphere.00233-25 (PMC12108089; doi:10.1128/msphere.00233-25)
Supplement: Supplemental figures — Figures S1 to S3. [file msphere.00233-25-s0004.pdf]

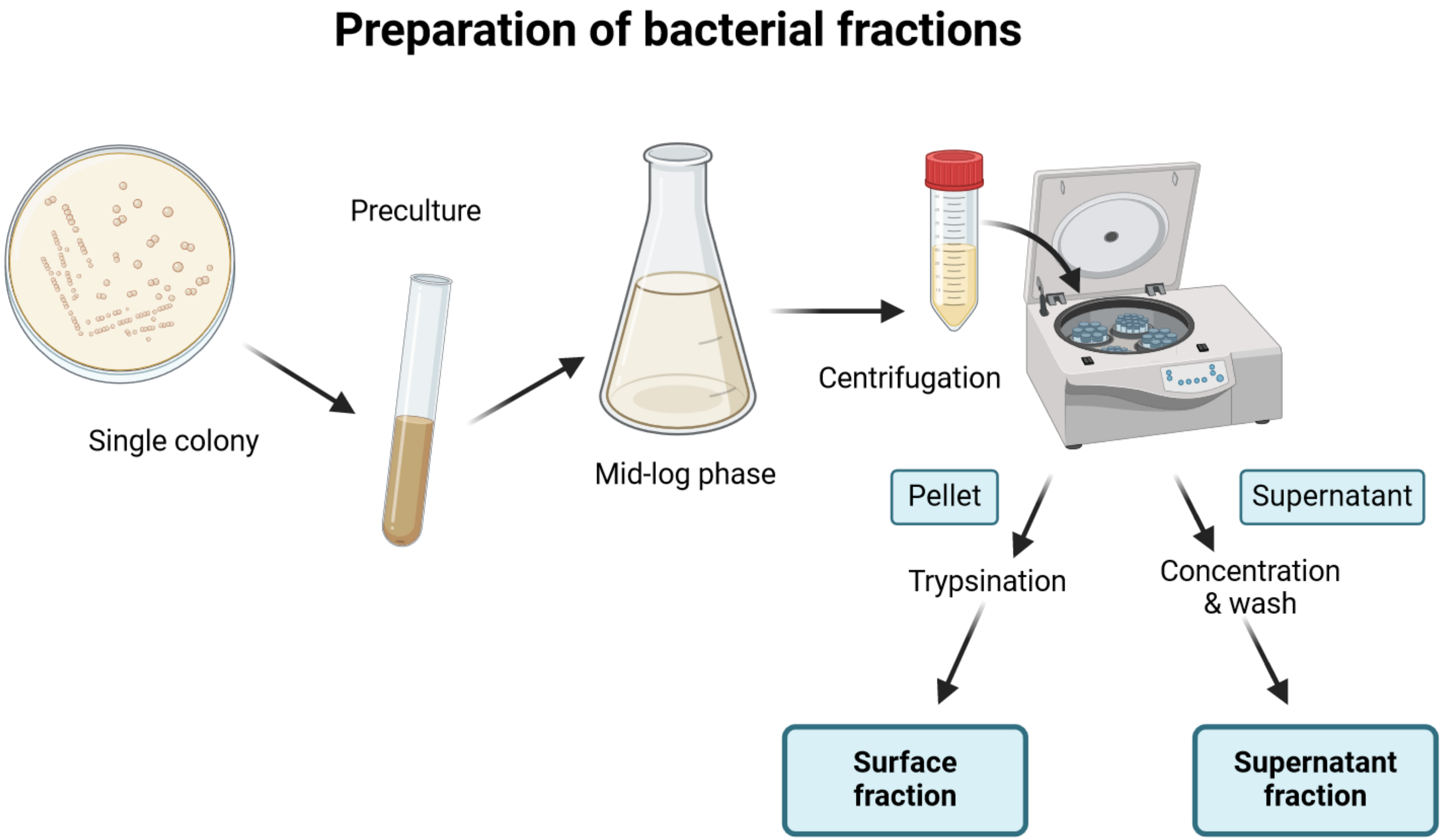

Supplementary figure 1. Schematic overview of the preparation of bacterial secreted and surface protein fractions. Created with BioRender.com.

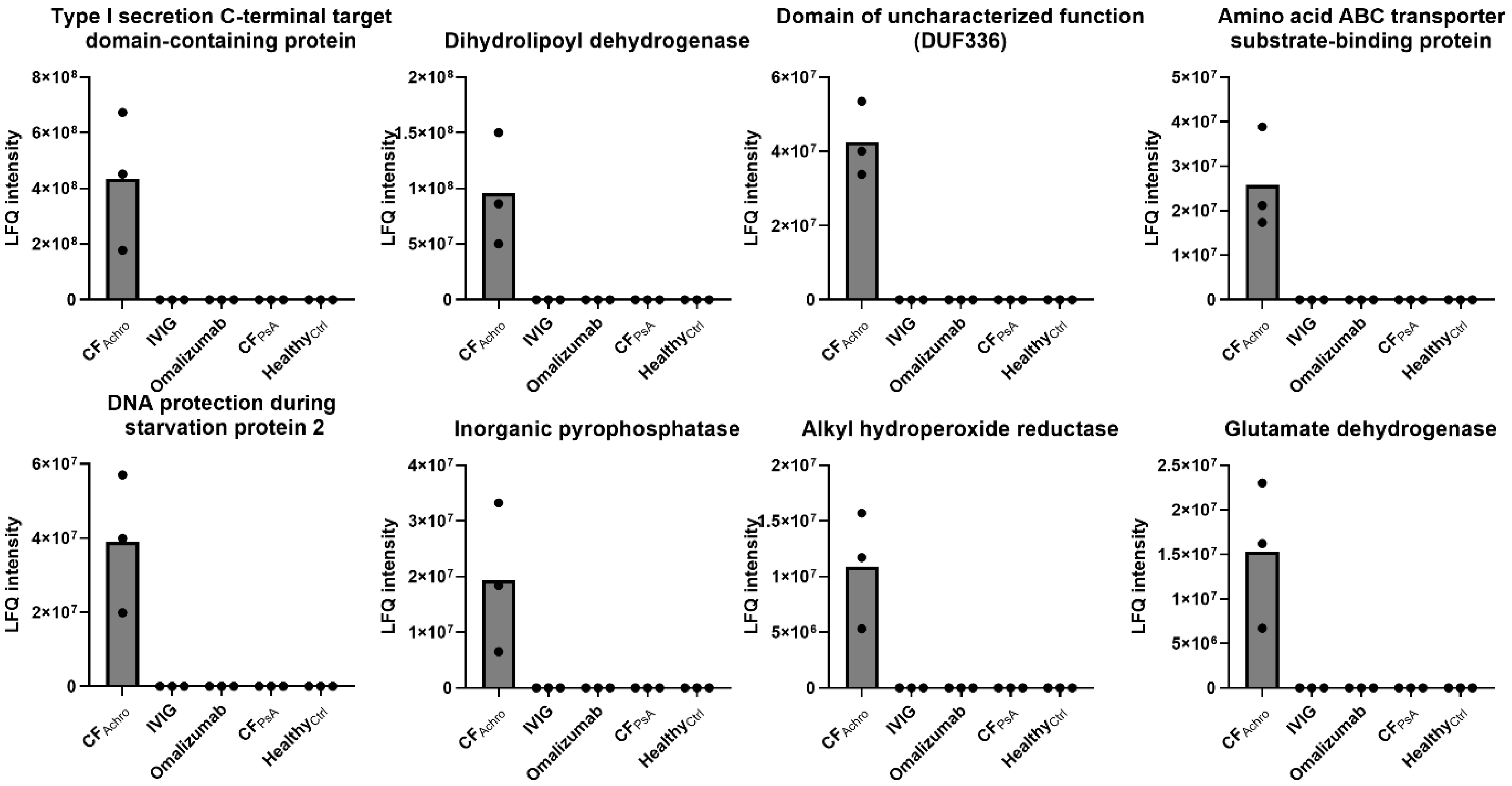

Supplementary figure 2. Raw intensities for eight *A. xylosoxidans* antigens after affinity purification on IgG from different sources.

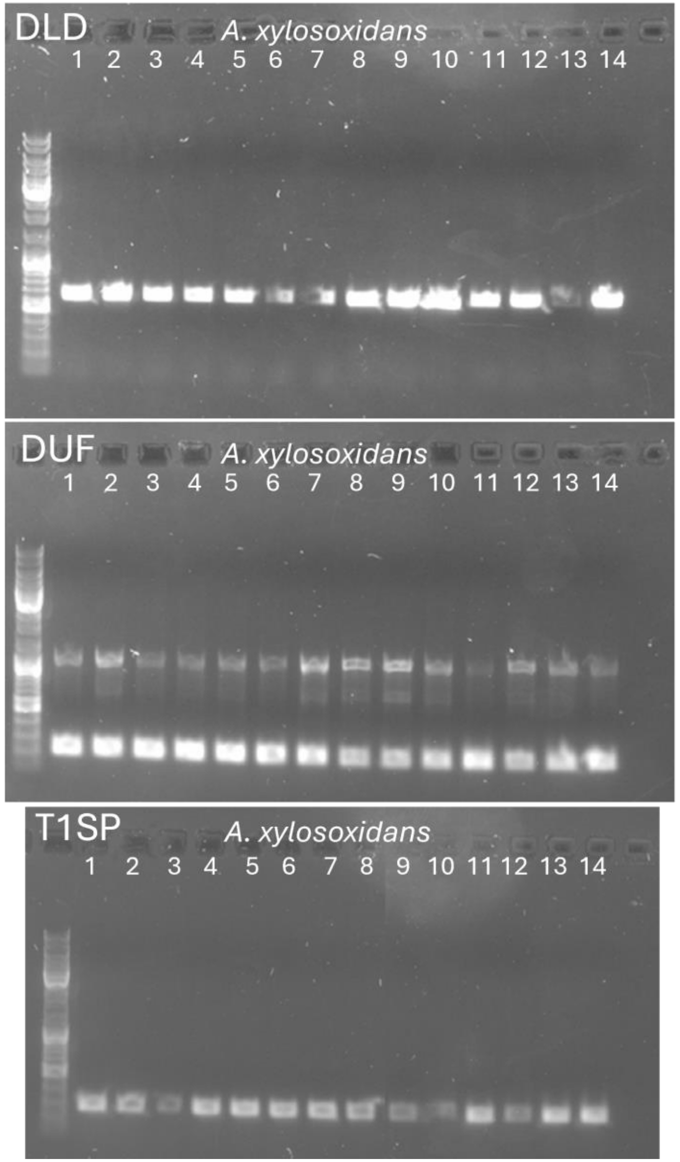

Supplementary figure 3. PCR of the genes corresponding to dihydrolipoyl dehydrogenase (DLD), Domain of uncharacterized function (DUF336) and Type I secretion C-terminal target domain-containing protein (T1S-DCP) in isolates of *A. xylosoxidans* (n=14).
