# Supplementary material for: Antibody-guided identification of Achromobacter xylosoxidans protein antigens in cystic fibrosis
Source: mSphere. 2025 Apr 29;10(5):e00233-25. doi: 10.1128/msphere.00233-25 (PMC12108089; doi:10.1128/msphere.00233-25)
Supplement: Supplemental legends — Supplemental file and figure legends. [file msphere.00233-25-s0005.pdf]

Supplemental file 1: Filtered MS results of bacterial protein contents.

Supplemental file 2: Filtered MS results of affinity purified antigenic candidates.

Supplemental file 3: BLAST and CLUSTALW alignment results of candidate antigen homology.

Supplementary figure 1: Schematic overview of the preparation of bacterial secreted and surface protein fractions.

Supplementary figure 2: Raw intensities for eight A. xylosoxidans antigens after affinity purification on IgG from different sources.

Supplementary figure 3: PCR of the genes corresponding to dihydrolipoyl dehydrogenase (DLD), Domain of uncharacterized function (DUF336) and Type I secretion C-terminal target domain-containing protein (T1S-DCP) in isolates of A. xylosoxidans (n=14).
